# Fractalis: a scalable open-source service for platform-independent interactive visual analysis of biomedical data

**DOI:** 10.1093/gigascience/giy109

**Published:** 2018-08-27

**Authors:** Sascha Herzinger, Valentin Grouès, Wei Gu, Venkata Satagopam, Peter Banda, Christophe Trefois, Reinhard Schneider

**Affiliations:** Luxembourg Centre for Systems Biomedicine, University of Luxembourg, Campus Belval, 7 av. des Hauts-Fourneaux, L-4362 Esch-sur-Alzette

**Keywords:** visualization, visual analytics, translational research, explorative analysis, federated analysis, web service

## Abstract

**Background:**

Translational research platforms share the aim of promoting a deeper understanding of stored data by providing visualization and analysis tools for data exploration and hypothesis generation. However, such tools are usually platform bound and are not easily reusable by other systems. Furthermore, they rarely address access restriction issues when direct data transfer is not permitted. In this article, we present an analytical service that works in tandem with a visualization library to address these problems.

**Findings:**

Using a combination of existing technologies and a platform-specific data abstraction layer, we developed a service that is capable of providing existing web-based data warehouses and repositories with platform-independent visual analytical capabilities. The design of this service also allows for federated data analysis by eliminating the need to move the data directly to the researcher. Instead, all operations are based on statistics and interactive charts without direct access to the dataset.

**Conclusions:**

The software presented in this article has a potential to help translational researchers achieve a better understanding of a given dataset and quickly generate new hypotheses. Furthermore, it provides a framework that can be used to share and reuse explorative analysis tools within the community.

## Background

In the field of translational research, we are facing an ever-growing amount of preclinical, clinical, OMICS, and mobile-sensor data that should be considered as a whole to understand the bigger picture of underlying diseases and biological processes. A platform that is able to store, link, and analyze the different data formats in an integrative manner is of urgent need. In recent years, several tools [1] have emerged in an attempt to solve these issues by providing a framework with standardized formats and tools for data-driven statistical analysis. Often performed before the time-consuming and computationally expensive hypothesis-driven analysis, the data-driven analysis helps researchers achieve a better understanding of the data and subsequently filter and generate new hypotheses. Some known examples of such translational research platforms are i2b2 [2], tranSMART [3], and cBioPortal [4]. These and other related platforms all share similar core functionalities that are responsible for analysis and visualization. Usually these internal analytical systems are complex constructs that require high maintenance due to changing data structures and requirements. This also explains why most, if not all, translational research platforms implement their own version of such systems. Statistical analysis scripts in those implementations usually make strong assumptions about the given data format, and the visual counterparts make similar strong assumptions about the user interface (UI), of which they are part of. This makes the implementation in a given platform substantially easier but at the same time highly platform dependent and, in most cases, unusable by other services. SmartR [5] is a concrete example for such a visual analytical system. It equips tranSMART with modern and interactive data analysis tools but suffers from the mentioned platform dependency, which makes the integration into other services very difficult. Shiny [6], Plotly [7], and Bokeh [8] are popular tools to address some aspects of this issue. They all make interactive visual analytics accessible to researchers with limited resources and, in some cases, can even be combined, as is the case with Shiny and Plotly. These tools operate at a lower level, which makes them customizable. However, in the case of data analysis, these tools difficult to share and reuse across different existing web applications with different data formats and application programming interfaces (APIs). Furthermore, none of the mentioned software tools directly address the data access restriction issue that often accompanies patient studies containing protected health information. Another approach is to provide researchers with a higher-level solution in the form of web applications with well-defined input formats, as shown in [9] and [10], that operate on a specific set of problems. This is a very powerful approach because it shifts the focus of the researcher to the data analysis instead of having to worry about data formats, analysis code, and APIs.

Fractalis is such a web application with focus on general hypothesis generation, data exploration, scalability, and ease of integration with existing platforms. It is capable of equipping existing translational research platforms with a powerful visualization component and an analytical system with federated data analysis abilities. Modern web technologies enable a dynamic and modern experience for the user, while a powerful distributed job pipeline ensures that the service is scalable and performant. Furthermore, the isolation of platform-specific code into a single layer makes it possible to extend Fractalis with support for virtually any translational research platform. This can lead to a significant reduction of resources invested in developing own visual analytical solutions.

## Findings

Building an external service for distributed data analysis alone would not solve the problem of platform dependency. APIs and data formats are likely unique to a given platform, so analysis scripts and visualizations based on the returned data would not be easily reusable. Instead, we split up the solution into two components. One is a web service that is capable of extracting data from foreign APIs and transforming them into an internal standard format, so given analysis scripts would always operate the same. The other component is a library that acts as a communication channel between the UI of the supported platform and the Fractalis back-end. It is also largely responsible for the visualization of the analysis results.

### The service

We used a Python web framework called Flask [11] as a base for our service. The main reason for that choice is the ability to use Python and R (via rpy2) natively for statistical computations. This also reduces the complexity of the application and massively improves the debugging capabilities by eliminating the need for an additional service such as RServe [12]. To avoid tight coupling with foreign platforms, we introduced a new concept called *Micro-ETL* within our service. Unlike usual ETLs (extract, transform, load) that migrate large parts of a database, these Micro-ETLs only migrate data that are currently required. The knowledge of what is required is relayed to the Fractalis service via a JavaScript library located within the foreign UI. Once this information reaches the service, a Micro-ETL factory decides which implementation can handle the migration based on the given information. What follows are the three major steps of every ETL:

1. The **e**xtraction of the data is usually done via REST API but can also involve other processes or protocols. Micro-ETLs contain all knowledge necessary to communicate with a given API or extract data by other means.
2. The **t**ransformation is the key to platform independence. It ensures that all incoming data are transformed to one of the internal standard formats (currently numerical data, categorical data, and array data); this makes all available analysis scripts reusable by other services that use Fractalis with similar data.
3. In the **l**oading step, the data are written to a nonpersistent cache whose location is tracked by Redis [13]. This prevents unnecessary data extraction in subsequent tasks. This is very important, given the fast-paced exploration that we want to provide.

Once the data are cached, we can perform statistical analyses on them and send the results, be it an HTML document, an image, or complex statistics, to the JavaScript library for further processing and visualization.

Some important challenges that visual analytics tools currently face are scalability in terms of parallel distributed job execution, federated analysis, and handling very large genomic datasets. In the following text, we introduce the Fractalis technology stack and describe how it handles these tasks.

**Figure 1:**
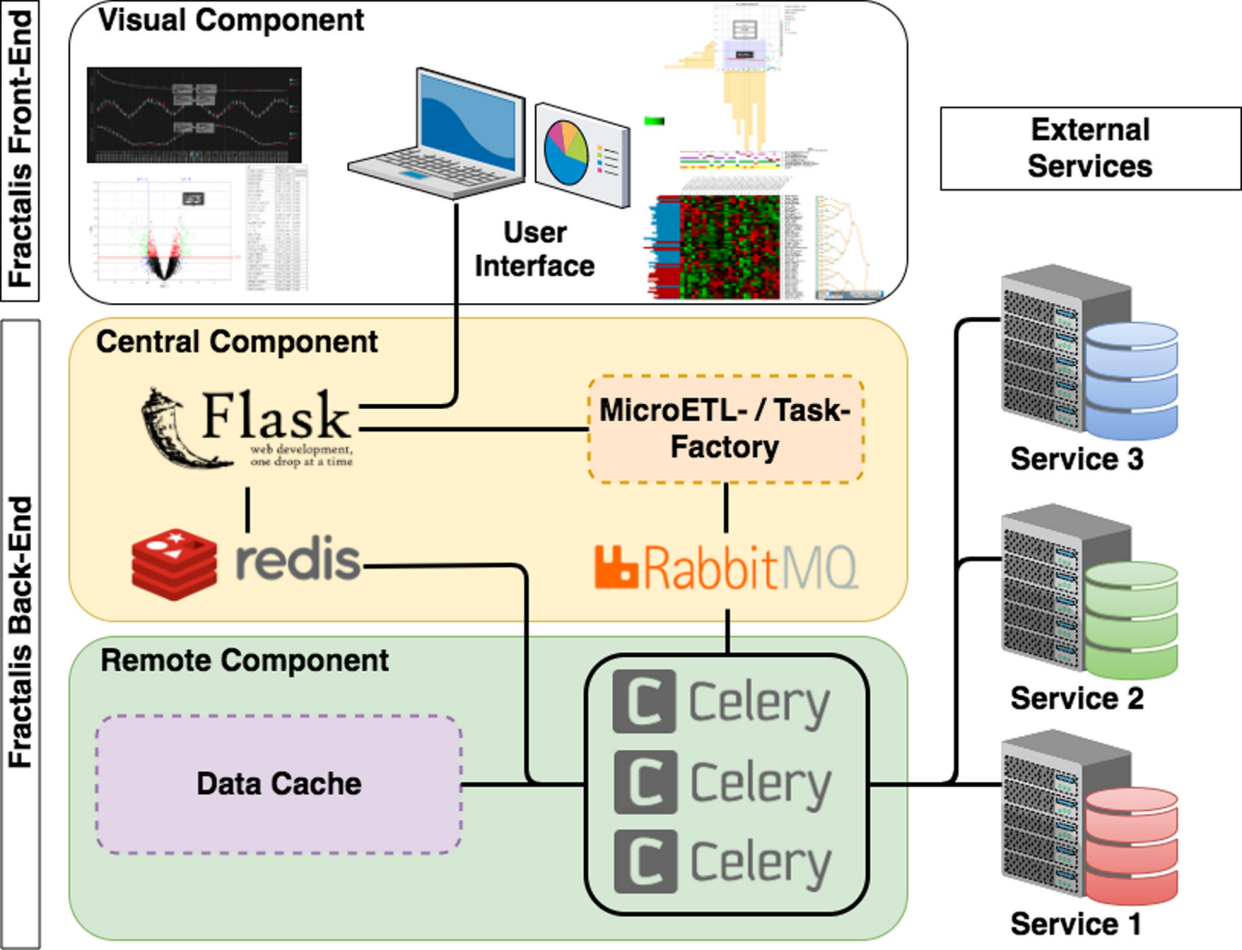
The Fractalis stack. Shown is a schematic view of the three major components: the visual component, which resides in the web browser and interacts with the user; the central server component, which manages the application states and handles job distribution; and the remote server component, which can be deployed remotely and handles the majority of the application workload.

The MicroETL and analysis stack mentioned before are supported by Celery [14] with RabbitMQ [15] as a message broker and Redis as a result and metadata store. A schematic of how these services are interconnected can be seen in Fig. 1, where it is shown that the back-end part of Fractalis is separated into a central component and a remote component. Celery allows us to spawn many computational nodes (workers) on different remote machines in order to move most of the workload out of the web service itself and enable support for a very large number of parallel requests by many users. One can also observe that the data cache resides in the remote component, which has no link to the central component. This design supports the federated analysis paradigm because it allows us to deploy workers in restrictive environments, where they can perform or relay analysis requests and only return statistics/results, not the data itself, to the central Redis result store, where they can be subsequently visualized.

The very same concept also enables the combination of small- to medium-sized phenotype/omics statistical analyses with large-scale genotype analyses, e.g., PCA, MDR, GWAS, and QTL. In such a scenario, Fractalis can handle the phenotype/omics data and relay analysis requests to frameworks such as Hail [16] that can analyze large-scale genotype data. Fractalis can then combine these results in a single visualization, which is often necessary to fully understand biological processes.

Developing new Micro-ETLs or analytics within Fractalis permits, but does not enforce, usage of the more advanced functionalities of the computational stack. For instance, it is possible to write a fully independent Python script that will be executed on a remote Celery worker without ever having heard of the technology. However, a more knowledgeable developer might want to queue several subsequent jobs or parallelize certain parts of the analytical process using the more advanced Celery interface. This is achieved by a design pattern known as *factory method*, which we used several times within the application to improve pluggability of new scripts and ETLs.

### The visualization library

**Figure 2:**
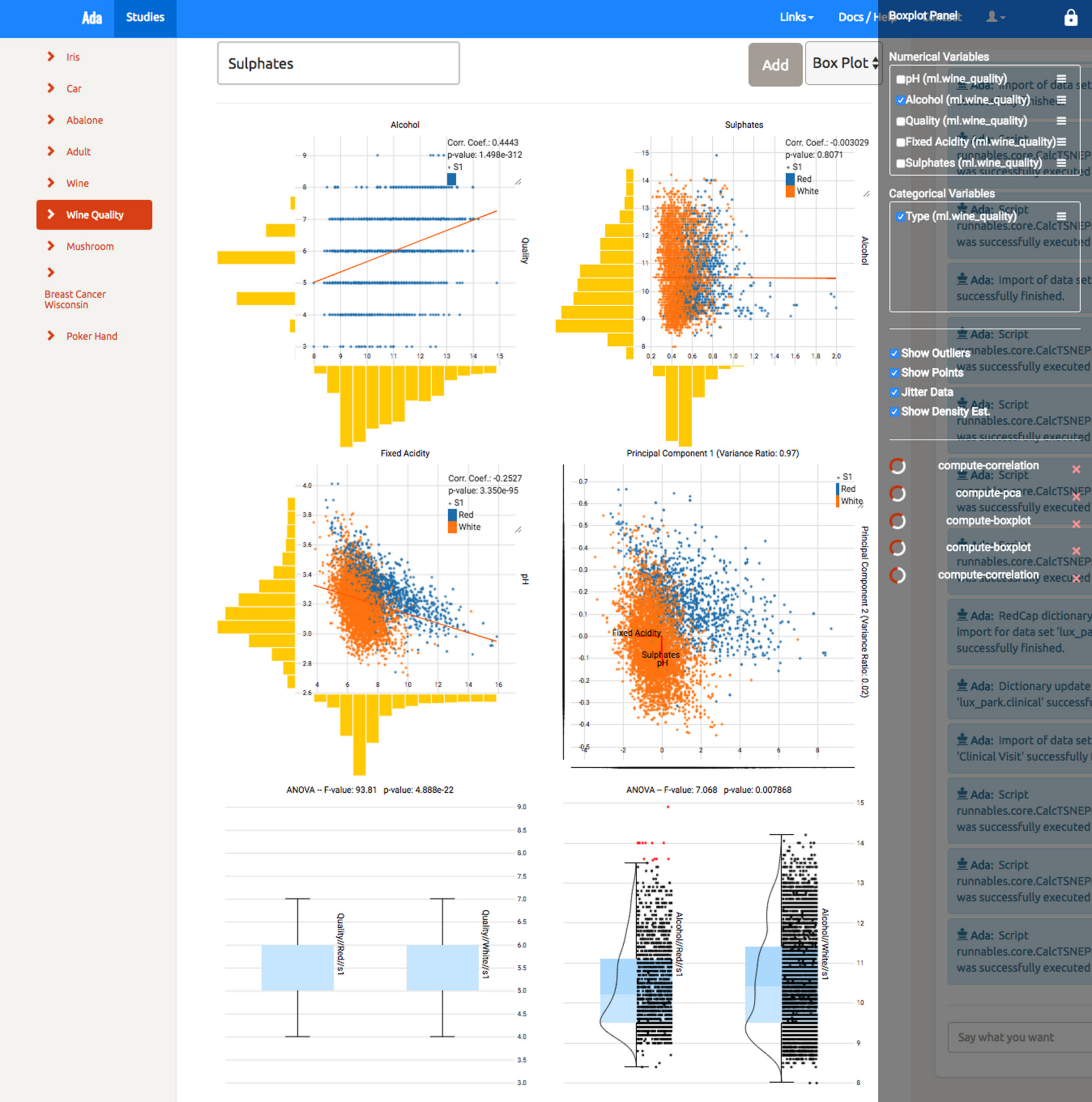
Fractalis in Ada. Shown is a self-hosted instance of Ada using Fractalis to display several statistics for the selected dataset. Notable is the native look of Fractalis within the existing UI, making the integration almost completely invisible to the user.

**Figure 3:**
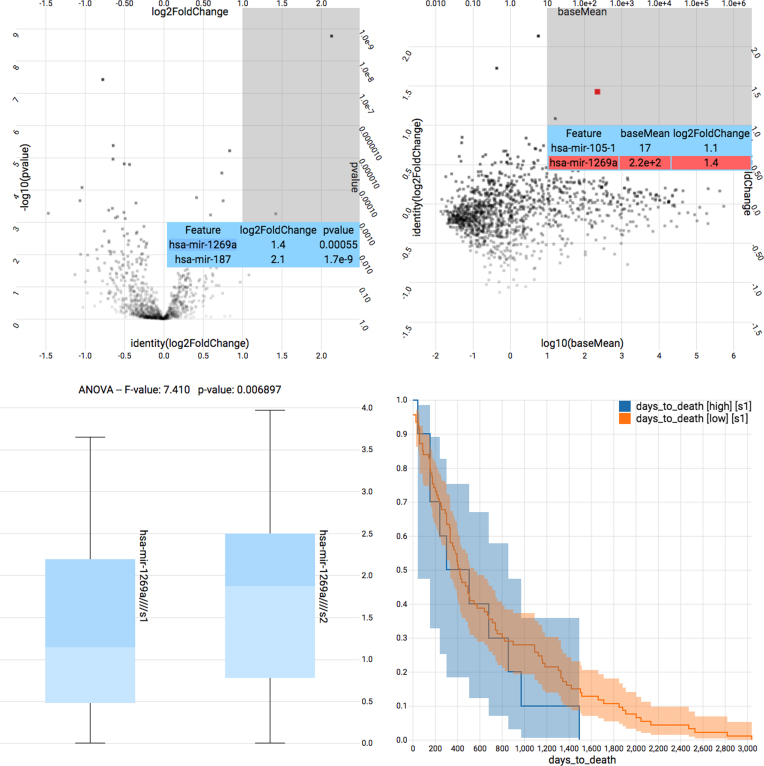
Fractalis pipeline demonstration. Shown are four Fractalis charts that show statistics based on the The Cancer Genome Atlas (TCGA)—colon adenocarcinoma (COAD) dataset. From left to right, top to bottom: a volcano plot using results of the R package DESeq2; an MA plot using results of the R package DESeq2; box plots and a one-way ANOVA group test; and a survival plot using the Kaplan-Meier estimator. The first three plots compare early-stage cancer with late-stage cancer. The last plot compares high read count of has-mir-1269a with low read count of has-mir-1269a.

The purpose of the front-end component is to provide a simple API that allows the integration into existing UIs and to render visualizations based on the statistics computed by the Fractalis service. In Figs. 2 and 3 one can see several examples of such visualizations: a scatter plot with correlation analysis and linear regression, a PCA, box plots with one-way analysis of variance (ANOVA), a volcano plot with differential expression analysis, and a survival analysis with Kaplan-Meier or Nelson-Aalen estimator.

The API is usually connected to platform-specific tools for patient subset selection and other methods for selecting variables of interest. This information is sent to the Fractalis service, where they trigger MicroETLs to prepare data for the analysis cache. This type of integration is illustrated in Fig. 2. The figure shows how we included Fractalis into another web-based data platform called Ada [17] to display statistics for a publicly available wine quality dataset (see [18]). To avoid potential conflicts with other libraries and the name scope of the parent application, we used native ES6/JavaScript components in combination with webpack [19], which compiles the entire project into a single scoped JavaScript file. Cross-browser compatibility and support for new or experimental features is ensured by Babel [20]. At the time of writing this manuscript, modern versions of Firefox, Chrome, and Safari were tested and supported. The charts have been created using Vue.js [21] together with a wide range of helper libraries. Reasons for this choice are the good documentation and the unopinionated nature of the framework, allowing for contributions by every moderately experienced JavaScript developer. We also extended Vue.js with Vuex [22] for the ability to have cross-component states. This is useful for mirroring the state of the server-side session or enabling the reaction of several components to a single event. In the case of Fractalis, we used this technology to connect all charts with each other such that a selection within a single chart (“brushing”) would inform all listeners about this event and subsequently trigger a recomputation and rerendering of the entire view. This is very useful for explorative analysis because it allows the researcher to select groups of interest and see instantly how statistics change in comparison. Simple examples for this are the comparison between case and control group or the exclusion of subjects with an age lower than 30 years. To fully understand how this technology works, we recommend a look into the videos and guides that are linked in the Supplementary Materials.

### Ensuring continuous reproducibility

Several measures have been taken to ensure reproducibility and ease of deployment when working with the Fractalis service. First, the code is properly documented and tested with roughly 250 unit and functional tests at the time of writing. These tests are executed for every code submission to our self-hosted GitLab repository and for every release. The release process is completely automated and requires no manual interaction. By pushing a new tag to the code repository, the continuous integration pipeline is instructed to build a new test environment, run all tests within this environment, and publish the build artifacts to their respective public repositories if all tests pass. Together, the artifacts are published to three repositories, namely, NPM [23] for the JavaScript library, PyPI [24] for the Python package, and Docker Hub for the Docker images [25]. To further simplify the deployment of Fractalis, we made use of the Docker Compose technology, which manages the service setup and network, including Redis, RabbitMQ, Nginx [26], Gunicorn [27], the Fractalis web service, and the Fractalis worker. In fact, the setup has become so simple that we encourage readers to follow the instructions in the Supplementary Materials and deploy Fractalis themselves.

#### Validation by example

We extensively discussed the technological aspects of the service presented in this manuscript, but so far have not mentioned a specific translational research use case. To demonstrate the usefulness of the tools described here, we selected a publication (Bu et al. 2015, [28]) with several plots that are based on analyses of the TCGA—COAD [29] dataset. In particular, we will focus on the miRNA quantification data and clinical data. All TCGA datasets are public and can be downloaded without registration from their repositories. The plots listed below can be found in [28] Fig. 1a, 1b, 1c, and 1e.

- a volcano plot based on a differential expression analysis of the microRNA quantification data,
- an MA plot based on the same data,
- a box plot with a group test for the difference between early- and late-stage expression of a certain microRNA, and
- a survival analysis based on the same microRNA between high and low read count of the same microRNA.

In the same order as above, the purpose of these analyses are:

- the discovery of up- or downregulated microRNA with high significance,
- making sure that microRNAs of potential interest are present in sufficiently abundance,
- testing whether there is a significant difference between early- and late-stage cancer for a certain microRNA, and
- testing whether the number of reads for a certain microRNA is correlated with the survival time of the patient.

To validate our analyses pipelines, we created every chart with Fractalis and checked if we came to conclusions that were similar to those of the publications' authors. It should be noted at this point that the TCGA—COAD dataset substantially grew (now 465 samples) since the article was published. Additionally, the authors did not describe their methods in detail, making a perfect reproduction very difficult, if not impossible. Nevertheless, the analyses results should be similar enough to make a valid comparison. Figure 3 shows the result of this comparison. We recorded a video of the process of creating these charts (Supplementary Materials) and included the dataset in the Docker image, so that interested readers can try to create the charts on their own. In the following, we describe our observations in detail.

First, we created the volcano plot (Supplementary Fig. S3a) by plotting the log2 fold change against the negative log10 fold change. These statistics where obtained by using the R DESeq2 package. We selected the sector within the chart with *P* ≤ 0.01 and log2 (fold change) ≥ 1.0, which revealed three microRNAs, namely, has-mir-1269a (*P* = 0.00055, log2(FC) = 1.4), has-mir-187 (*P* = 1.7e-7, log2(FC) = 2.1), and has-mir-934 (*P* = 0.0035, log2(FC) = 1.7). Subsequently, we created a MA plot (Supplementary Fig. S3b) using the same results, but this time plotting log10(baseMean) against log2(FC) and selected the sector within the chart with baseMean ≥ 10 and log2(FC) ≥ 1.0. This revealed has-mir-105-1 (baseMean = 17, log2(FC) = 1.1) and has-mir-1269a (baseMean = 2.2e+2, log2(FC) = 1.4). Intersecting this result with the previous one leaves has-mir-1269a as the only microRNA with sufficient abundance, significance, and noteworthy fold change between early- and late-stage cancer. So far, our results are in alignment with the findings reported by the authors. We singled out the has-mir-1269a row from the data matrix and compared the two groups within a box plot chart (Supplementary Fig. S3c) with log10 transformed data. The one-way ANOVA reported F = 7.410 with *P* = 0.0069. Our box plots are almost identical to the reported ones, but our group test has a slightly better *P* value, likely due to the additional samples in the dataset. Finally, we compared samples with high has-mir-1269a read count (reads >1,500) with low has-mir-1269a read count (reads <1,500) by performing a survival analysis with a Kaplan-Meier estimator. We only included patients with an observed death event in both groups, but our results are in rough agreement with the observations of Bu et al., who likely considered the entire cohort. It should be noted here that the group with high read has-mir-1269a count is very small, which is highlighted by the very large confidence intervals. All charts that are shown can be generated in a matter of seconds or minutes (differential expression analysis is computationally expensive) within Fractalis.

### Latency benchmarks

The Fractalis back-end can be horizontally scaled by using a distributed worker architecture. As mentioned above, this particular point also allows for federated analysis, which is why it is of particular interest how well this architecture performs when there is a large physical distance between the central and remote nodes. For this purpose, we used Google Cloud to deploy worker nodes in the United Kingdom and the United States, with a central node in Germany and the actual user interface in Luxembourg. To get a baseline, we deployed the same setup in our intranet so we can approximate the introduced latency. To avoid a potential bias due to different hardware specifications, we limited this test to the execution of a simple correlation analysis with linear regression between two variables within the TCGA—COAD dataset from above. In this way, only the network latency between the different services would be measured, not how well the central processing unit and random access memory perform. It should also be noted that the current front-end is polling in intervals for the result, so the measured latency will be higher than the actual latency. Table 1 lists the outcome of the three different setups. Unsurprisingly, the intranet deployment is the fastest, with an average of 88 ms between submitting an analysis and receiving the results. This is the most common form of deployment and will be sufficient for any potential use case targeted by Fractalis. Moving the central component to a server in Frankfurt, Germany, and the worker to a server in London, UK, resulted in an expected increase in the latency. With 207 ms on average, this latency is hardly noticeable, making a large distributed network within Europe more than feasible. Moving the worker to a server in South Carolina, USA introduced a noticeable lag of, on average, 792 ms. While this is a 9-fold increase in comparison to our intranet baseline, the delay is still in the subsecond range. Most explorative analyses with a moderately sized dataset will run for several seconds, making a subsecond delay in most cases negligible.

**Table 1:** Fractalis distributed pipeline benchmark

Worker location	Ping, ms	1st meas., ms	2nd meas., ms	3rd meas., ms	Avg., ms
Intranet	<1	92	84	88	88
London, UK (Google Cloud)	20	222	200	199	207
South Carolina, USA (Google Cloud)	102	794	794	789	792

The table shows the time past between submitting an analysis and receiving the results. All results include the time needed to prepare the data for analysis, the computation of the correlation statistics, the sending of the results, and the latency/overhead introduced by the communication between the service components. The Ping column shows the base latency by pinging the server from our location in Luxembourg.

### Discussion and outlook

Here, we presented a framework for explorative visual analysis of biomedical data. Major features include easy integration into almost all existing translational research platforms and the heavily distributed architecture that permits high scalability and enables analysis federation functionality. For platforms with little or no explorative data analysis, Fractalis is a real alternative to developing an individual solution and can save development resources and give researchers access to many useful tools for hypothesis generation. Furthermore, individual visualizations and analyses can easily be shared with other researchers, even if the underlying data warehouse platform is different.

We demonstrated the hypothesis-generation capabilities by quickly generating several charts and comparing them with an existing publication and recorded the work for educational purpose.

A few standard analyses have been included in Fractalis to showcase the software and gather an initial user base. Some examples are survival analyses, box plots, scatter plots with correlation analysis and linear regression, volcano plots and heat maps with differential expression analysis performed by the R packages limma and DESeq2, and principle component analyses.

New developments will take user feedback into account and prioritize the implementation of much needed analyses and features. In a similar fashion, support for new platforms in the form of new MicroETLs will follow. At the time of writing, Fractalis is being integrated into the following three platforms:

1. tranSMART 17.1 including the new data model, API, and UI;
2. Ada [manuscript in preparation], an internally developed data integration service; and
3. i2b2-tranSMART [manuscript in preparation], a platform developed by the recently merged i2b2 Foundation and tranSMART Foundation. Note: This platform is *very* different from 1.

Future developments might include a stand-alone version of Fractalis, which runs on the computer of the researcher to permit uploading and analyzing local files via the user interface instead of extracting it from an external service.

## Availability and requirements

Project name: Fractalis

RRID:SCR_016362

Project home page: https://fractalis.lcsb.uni.lu/

Operating systems: All Docker supported operating systems (e.g., most Linux distributions, MacOS, MS Windows)

Programming languages: Python, JavaScript

Requirements: Python 3.6 or higher, a recent version of Chrome, Firefox, or Safari

License: Apache 2.0

## Availability of supporting data

The dataset supporting the results presented here is available at https://portal.gdc.cancer.gov/repository. Snapshots of the code and other supporting data are also openly available in the *GigaScience* repository, GigaDB [30].

## Additional files

Supplementary Material.docx

## Abbreviations

ANOVA: analysis of variance; API: Application Programming Interface; COAD: colon adenocarcinoma; ETL: extract transform load; TCGA: The Cancer Genome Atlas; UI: user interface.

## Competing interests

The authors declare that they have no competing interests.

## Funding

Acknowledgement is made for support by the Fonds Nationale de la Recherche Luxembourg, through the National Centre of Excellence in Research on Parkinson's Disease (NCER13/BM/11264123). This work was partially funded through the contribution of the Luxembourg Ministry of Higher Education and Research Towards the Luxembourg ELIXIR Node.

## Author contributions

S.H. planned and executed the project. V.G. contributed knowledge to the architecture. P.B. is a very early adopter of Fractalis and his feedback influenced the development process. C.T. provided the necessary infrastructure for a large-scale setup and helped with the deployment. W.G., V.S., and R.S. are senior researchers who helped to understand the current translational research landscape and its challenges. All authors read the manuscript and provided feedback.

## Supplementary Material

GIGA-D-18-00166_Original_Submission.pdf

GIGA-D-18-00166_Revision_1.pdf

Response_to_Reviewer_Comments_Original_Submission.pdf

Reviewer_1_Report_(Original_Submission) -- Konstantinos Krampis 05/12/2018 Reviewed

Reviewer_2_Report_(Original_Submission) -- Benjamin Glicksberg 05/27/2018 Reviewed

Reviewer_1_Report_Revision_1 -- Konstantinos Krampis 07/31/2018 Reviewed

Reviewer_2_(Report_Revision_1) -- Benjamin Glicksberg 08/12/2018 Reviewed

Supplement File
