# Supplementary material for: Fractalis: a scalable open-source service for platform-independent interactive visual analysis of biomedical data
Source: Gigascience. 2018 Aug 27;7(9):giy109. doi: 10.1093/gigascience/giy109 (PMC6143733; doi:10.1093/gigascience/giy109)
Supplement: GIGA-D-18-00166_Original_Submission.pdf [file giy109_giga-d-18-00166_original_submission.pdf]

GigaScience

Fractalis: A scalable open-source service for platform-independent interactive visual analysis of biomedical data

--Manuscript Draft--

**Manuscript Number:**	GIGA-D-18-00166
**Full Title:**	Fractalis: A scalable open-source service for platform-independent interactive visual analysis of biomedical data
**Article Type:**	Technical Note
**Funding Information:**	Fonds Nationale de la Recherche (FNR) Luxembourg (NCER13/BM/11264123) — Not applicable
**Abstract:**	Background: Translational research platforms share the aim to promote a deeper understanding of stored data by providing visualization and analysis tools for data exploration and hypothesis generation. However, such tools are usually platform-bound and are not easily reusable by other systems. Furthermore, they rarely address access restriction issues when direct data transfer is not permitted. In this article we present an analytical service that works in tandem with a visualization library to address these problems.<br>Findings: Using a combination of existing technologies and a platform-specific data abstraction layer we developed a service that is capable of providing existing web-based data warehouses and repositories with platform-independent visual analytical capabilities. The design of this service also enables federated data analysis by bringing the analysis to the data and only present results to the researcher.<br>Conclusion: The software presented in this article has the potential to help translational researchers achieve a better understanding of a given dataset and quickly generate new hypothesis. Furthermore, it provides a freamework that can be used to share and reuse explorative analysis tools within the community.
**Corresponding Author:**	Sascha Herzinger<br>LCSB - University of Luxembourg<br>LUXEMBOURG
**Corresponding Author Secondary Information:**	
**Corresponding Author's Institution:**	LCSB - University of Luxembourg
**Corresponding Author's Secondary Institution:**	
**First Author:**	Sascha Herzinger, MSc
**First Author Secondary Information:**	
**Order of Authors:**	Sascha Herzinger, MSc
	Valentin Grouès, MSc
	Wei Gu, PhD
	Venkata Satagopam, MSc
	Peter Banda, PhD
	Christophe Trefois, PhD
	Reinhard Schneider, PhD
**Order of Authors Secondary Information:**	
**Additional Information:**	
**Question**	**Response**
Are you submitting this manuscript to a special series or article collection?	No

Powered by Editorial Manager® and ProduXion Manager® from Aries Systems Corporation

**Experimental design and statistics**<br><br>Full details of the experimental design and statistical methods used should be given in the Methods section, as detailed in our Minimum Standards Reporting Checklist. Information essential to interpreting the data presented should be made available in the figure legends.<br><br>Have you included all the information requested in your manuscript?	Yes
**Resources**<br><br>A description of all resources used, including antibodies, cell lines, animals and software tools, with enough information to allow them to be uniquely identified, should be included in the Methods section. Authors are strongly encouraged to cite Research Resource Identifiers (RRIDs) for antibodies, model organisms and tools, where possible.<br><br>Have you included the information requested as detailed in our Minimum Standards Reporting Checklist?	Yes
**Availability of data and materials**<br><br>All datasets and code on which the conclusions of the paper rely must be either included in your submission or deposited in publicly available repositories (where available and ethically appropriate), referencing such data using a unique identifier in the references and in the "Availability of Data and Materials" section of your manuscript.<br><br>Have you have met the above requirement as detailed in our Minimum Standards Reporting Checklist?	Yes

Click here to download Manuscript Manuscript.docx

Fractalis: A scalable open-source service for platform-independent interactive visual analysis of biomedical data

Sascha Herzinger sascha.herzinger@uni.lu (correspondence)

Valentin Grouès valentin.groues@uni.lu

Wei Gu wei.gu@uni.lu

Venkata Satagopam venkata.satagopam@uni.lu

Peter Banda peter.banda@uni.lu

Christophe Trefois christophe.trefois@elixir-luxembourg.org

Reinhard Schneider reinhard.schneider@uni.lu

Luxembourg Centre for Systems Biomedicine, University of Luxembourg, Belvaux, Luxembourg.

Abstract

Background: Translational research platforms share the aim to promote a deeper understanding of stored data by providing visualization and analysis tools for data exploration and hypothesis generation. However, such tools are usually platform-bound and are not easily reusable by other systems. Furthermore, they rarely address access restriction issues when direct data transfer is not permitted. In this article we present an analytical service that works in tandem with a visualization library to address these problems.

Findings: Using a combination of existing technologies and a platform-specific data abstraction layer we developed a service that is capable of providing existing web-based data warehouses and repositories with platform-independent visual analytical capabilities. The design of this service also enables federated data analysis by bringing the analysis to the data and only present results to the researcher.

Conclusion: The software presented in this article has the potential to help translational researchers achieve a better understanding of a given dataset and quickly generate new hypothesis. Furthermore, it provides a freamework that can be used to share and reuse explorative analysis tools within the community.

Keywords

visualization, visual analytics, translational research, explorative analysis, federated analysis, web service

Background

In the field of translational research, we are facing an ever-growing amount of pre-clinical, clinical, OMICS, and mobile-sensor data that has to be considered as a whole to understand

the bigger picture of underlying diseases and biological processes. A platform that is able to store, link, and analyze the different data formats in an integrative manner is of urgent need. In recent years several tools [1] have emerged that attempt to solve these issues by providing a framework with standardized formats and tools for *data-driven* statistical analysis. Often performed before the time-consuming and computationally expensive *hypothesis-driven* analysis, the data-driven analysis helps researchers achieve a better understanding of the data and subsequently filter and generate new hypothesis. Some known examples of such translational research platforms are i2b2 [2], tranSMART [3], and cBioPortal [4]. These and other related platforms all share similar core functionalities that are responsible for analysis and visualisation. Usually these internal analytical systems are complex constructs that require high maintenance due to changing data structures and requirements. This also explains why most, if not all, translational research platforms implement their own version of such systems. Statistical analysis scripts in those implementations usually make strong assumptions about the given data format, and the visual counterparts make similar strong assumptions about the user interface (UI), of which they are part of. This makes the implementation in a given platform substantially easier, but at the same time highly platform-dependent, and in most cases unusable by other services. SmartR [5] is a concrete example for such a visual analytical system. It equips tranSMART with modern and interactive data analysis tools, but suffers from the mentioned platform-dependency, which makes the integration into other services very difficult. Shiny [6], Plotly [7], and Bokeh [8] are popular tools to address some aspects of this issue. They all make interactive visual analytics accessible to researchers with limited resources and time. However, the missing data abstraction layer makes implementations solely based on these frameworks difficult to share and reuse in other services. Furthermore, none of the mentioned software tools directly addresses the data access restriction issue that often accompanies patient studies.

To address these problems, we developed Fractalis. Fractalis is a highly scalable and reusable service that works alongside existing research platforms to equip them with a visualization component and an analytical system with federated data analysis capabilities.

Findings

Building an external service for distributed data analysis alone would not solve the problem of platform-dependency. APIs (Application Programming Interfaces) and data formats are likely unique to a given platform, so analysis scripts and visualizations based on the returned data would not be easily reusable. Instead we split up the solution into two components. One is a web service that is capable of extracting data from foreign APIs and transforming them into an internal standard format, so given analysis scripts would always operate the same. The other component is a library that acts as a communication channel between the user interface (UI) of the supported platform and the Fractalis back-end. It is also largely responsible for the visualisation of the analysis results.

The service

We used a Python web framework called Flask [9] as a base for our service. The main reason for that choice is the ability to use Python and R (via rpy2) natively for statistical computations. This also reduces the complexity of the application and massively improves the debugging capabilities by eliminating the need for an additional service like RServe [10]. To avoid tight coupling with foreign platforms we introduced a new concept called *Micro-ETL* within our service. Unlike usual ETLs (Extract, Transform, Load) that migrate large parts of a database, these Micro-ETLs only migrate data that are currently required. The knowledge of what is required is relayed to the Fractalis service via a Javascript library located within the foreign

UI. Once this information reaches the service, a Micro-ETL factory decides which implementation can handle the migration based on the given information. What follows are the three major steps of every ETL:

- The **e**xtraction of the data is usually done via REST API but can also involve other processes or protocols. Micro-ETLs contain all knowledge necessary to communicate with a given API or extract data by other means.
- The **t**ransformation is the key to platform independence. It ensures that all incoming data are transformed to one of the internal standard formats, that makes all available analysis scripts reusable by other services that use Fractalis with similar data.
- In the **l**oading step, the data are written to a non-persistent cache, whose location is tracked by Redis [11]. This avoids unnecessary data extraction in subsequent tasks. This is very important, given the fast-paced exploration that we want to provide.

Once the data are cached, we can perform statistical analyses on them and send the results, be it a HTML document, an image, or complex statistics to the Javascript library for further processing and visualization.

Some important challenges that visual analytics tools currently face are scalability in terms of parallel distributed job execution, federated analysis, and handling very large genomic data sets. In the following we introduce the Fractalis technology stack and describe how it handles these tasks.

The MicroETL and analysis stack mentioned before is supported by Celery [12] with RabbitMQ [13] as a message broker and Redis as result and metadata store. A schematic illustration of how these services are interconnected can be seen in Figure 1, where it is shown that the back-end part of Fractalis is separated into a central and remote component. Celery

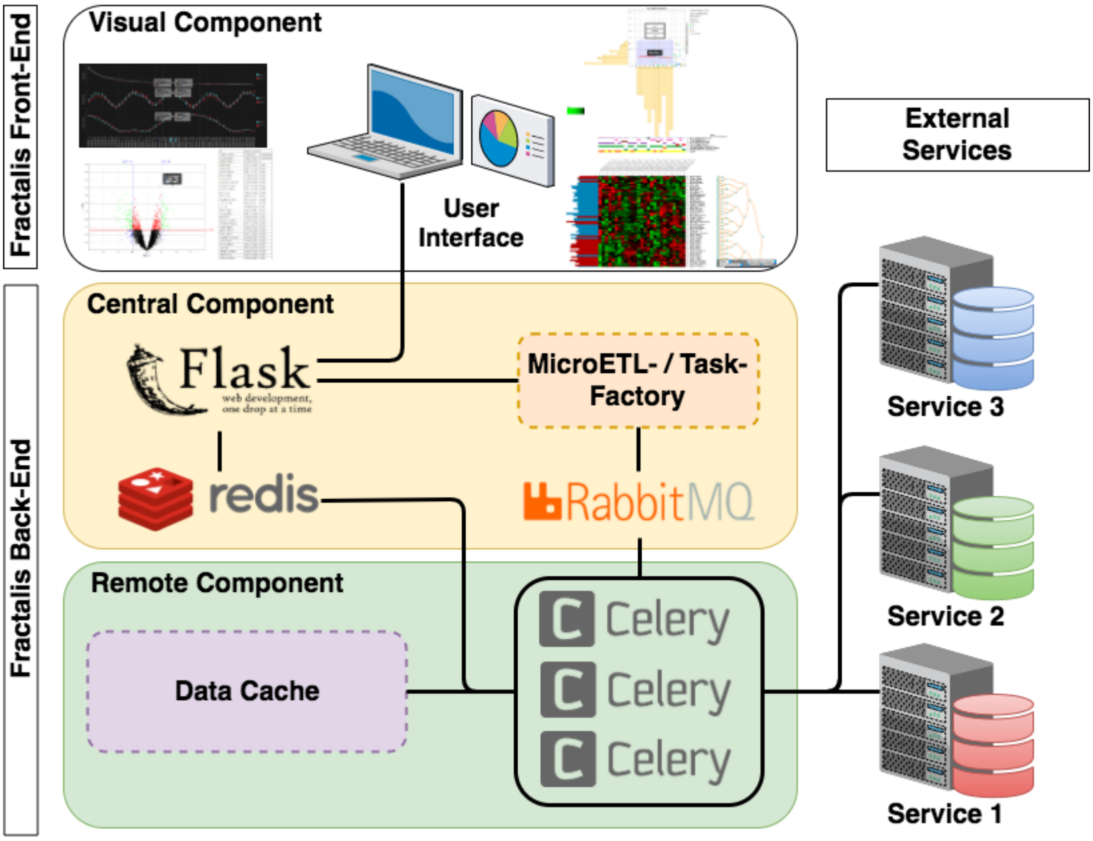

Figure 1 The Fractalis stack. Shown is a schematic view of the three major components: 1) The visual component, which resides in the web browser and interacts with the user. 2) The central server component which manages the application states and handles job distribution. 3) The remote server component, which can be deployed remotely and handles the majority of the application workload.

allows us to spawn many computational nodes (workers) on different remote machines in order to move most of the workload out of the web service itself and enable support for a very large number of parallel requests by many users. One can also observe that the data cache resides in the remote component, which has no link to the central component at all. This design supports the federated analysis paradigm because it allows us to deploy workers in restrictive environments, where they can perform or relay analysis requests and only return statistics/results, not the data itself, to the central Redis result store, and subsequently be visualised. The very same concept also enables analysis of extremely large genomic data sets. In such a scenario, Fractalis can handle the smaller phenotypical data and relay analysis requests based on these to a computational node that is located in close proximity to a framework like Hail [14] that can store and analyse genomic data. This is also a good example

of how Fractalis can use different data sources and data types and combine them in a single visualisation, which is often necessary to fully understand biological processes.

Developing new Micro-ETLs or analytics within Fractalis permits, but does not enforce, usage of the more advanced functionalities of the computational stack. For instance, it is possible to write a fully independent Python script that will be executed on a remote Celery worker without ever having heard of the technology. However, a more knowledgeable developer might want to queue several subsequent jobs or parallelize certain parts of the analytical process using the more advanced Celery interface. This is achieved by a design pattern known as *factory method pattern*, which we used several times within the application, to improve pluggability of new scripts and ETLs.

The visualization library

The purpose of the front-end component is to provide a simple API that allows the integration into existing user interfaces, and to render visualizations based on the statistics computed by the Fractalis service. The API is usually connected to platform specific tools for patient subset selection and other methods for selecting variables of interest. This information is sent to the Fractalis service, where they trigger MicroETLs to prepare data for the analysis cache. How such an integration can look like is shown in Figure 2. Here can be seen how we included Fractalis into another web-based data platform called Ada [15] to display statistics for a publicly available dataset [16]. To avoid potential conflicts with other libraries and the name scope of the parent application we used native ES6/Javascript components in combination with webpack [17], which compiles the entire project into a single scoped Javascript file. Cross-browser compatibility and support for new or experimental features is ensured by Babel [18]. At the time of writing of this manuscript, modern versions of Firefox, Chrome, and Safari are all tested and supported. The charts have been created using Vue.js [19] together with a wide

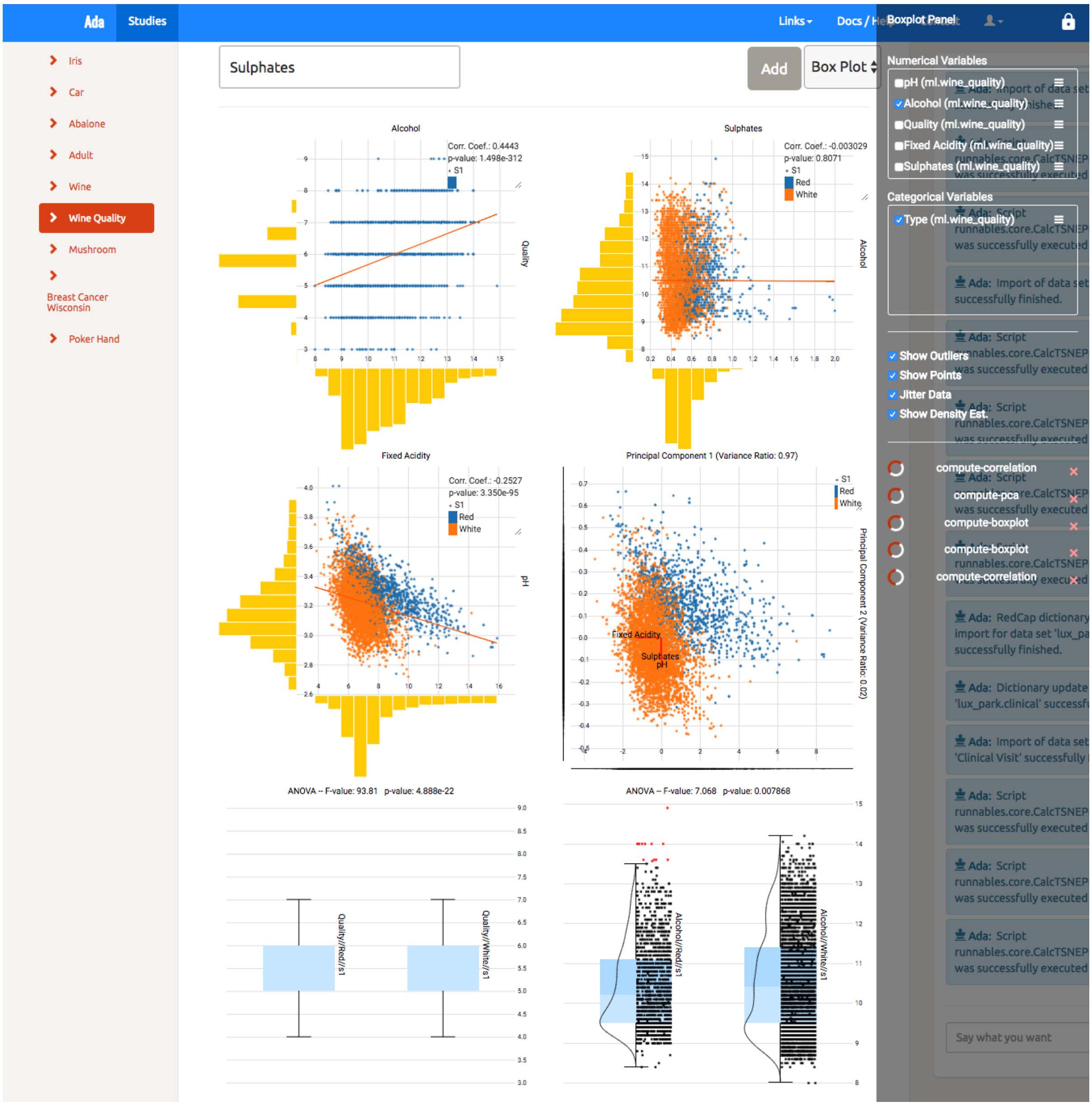

Figure 2 Fractalis in Ada. Shown is a self-hosted instance of Ada using Fractalis to display several statistics for the selected dataset. Notable is the native look of Fractalis within the existing user interface, making the integration almost completely invisible to the user.

range of helper libraries. Reasons for this choice are the good documentation and the unopinionated nature of the framework, allowing for contributions by every moderate experienced Javascript developer. We also extended Vue.js with Vuex [20] for the ability to have cross-component states. This is useful for mirroring the state of the server-side session or enabling the reaction of several components to a single event. In the case of Fractalis, we used this technology to connect all charts with each other in a way such that a selection within a single chart ("brushing") would inform all listeners about this event and subsequently trigger

a re-computation and re-rendering of the entire view. For explorative analysis this is very useful, because it allows the researcher to select groups of interest and see instantly how statistics change in comparison. Simple examples for this are the comparison between case and control group, or the exclusion of subjects with an age lower than 30. To fully understand how this technology works we recommend a look into the videos and guides that are linked in the supplementary materials.

Ensuring continuous reproducibility

Several measures have been taken to ensure reproducibility and ease of deployment, when working with the Fractalis service. First of all, the code is properly documented and tested with roughly 250 unit and functional tests at the time of writing. These tests are executed for every code submission to our self-hosted GitLab repository and for every release. The release process is completely automated and requires no manual interaction. By pushing a new tag to the code repository, the continuous integration (CI) pipeline is instructed to build a new test environment, run all tests within this environment, and publish the build artifacts to their respective public repositories if all tests pass. Altogether the artifacts are published to three repositories, namely NPM [21] for the Javascript library, PyPI [22] for the Python package, and Docker Hub for the Docker images [23]. To even further simplify the deployment of Fractalis we made use of the Docker Compose technology, which manages the service setup and network including Redis, RabbitMQ, Nginx [24], Gunicorn [25], the Fractalis web service, and the Fractalis worker. In fact, the setup has become so simple that we encourage the readers to follow the instructions in the supplementary materials and deploy Fractalis themselves.

Outlook

A few standard analyses have been included to showcase the software and gather an initial user base. New developments will take user feedback into account and prioritize the implementation of much needed analyses and features. In a similar fashion, support for new platforms in form of new MicroETLs will follow. At the time of writing, we are working on the integration for three different platforms:

1. tranSMART 17.1 including the new data model, API, and UI
2. Ada, an internally developed data integration service
3. i2b2-tranSMART [manuscript in preparation], a platform developed by the recently merged i2b2 Foundation and tranSMART Foundation. Note: This platform is *very* different from 1.

Further future developments might include a stand-alone version of Fractalis, which runs on the computer of the researcher to permit uploading and analyzing local files via the user interface instead of extracting it from an external service.

Availability and requirements

Project name: Fractalis

Project home page: https://fractalis.lcsb.uni.lu/

Operating systems: All Docker supported operating systems (e.g. most Linux distributions, MacOS, MS Windows)

Programming languages: Python, Javascript

Requirements: Python 3.4 or higher, a recent version of Chrome, Firefox, or Safari

License: Apache 2.0

Availability of supporting data

Not applicable

List of abbreviations

API – Application Programming Interface

CI – Continuous Integration

ETL – Extract Transform Load

UI – User Interface

Ethics approval and consent to participate

Not applicable

Consent for publication

Not applicable

Competing interests

The authors declare that they have no competing interests.

Funding

Acknowledgement is made for support by the Fonds Nationale de la Recherche (FNR) Luxembourg, through the National Centre of Excellence in Research (NCER) on Parkinson's disease, NCER13/BM/11264123.

This work was partially funded through the contribution of the Luxembourg Ministry of Higher Education and Research towards the Luxembourg ELIXIR Node.

Authors' contributions

SH planned and executed the project. VG contributed their knowledge to the architecture. PB is a very early adopter of Fractalis and their feedback influenced the development process. CT provided the necessary infrastructure for a large-scale setup and helped with the deployment. WG, VS, RS are senior researchers that helped to understand the current translational research landscape and its challenges. All authors read the manuscript and provided feedback.

Acknowledgements

The authors would like to thank their colleagues and the Reproducible Research Results (R3) team of the Luxembourg Centre for Systems Biomedicine for support of the project and for promoting reproducible research.

References

[1] Canuel V, Rance B, Avillach P, Degoulet P, Burgun A. Translational research platforms integrating clinical and omics data: A review of publicly available solutions. Brief Bioinform. 2015;16:280–90.

[2] Murphy SN, Weber G, Mendis M, Gainer V, Chueh HC, Churchill S, et al. Serving the enterprise and beyond with informatics for integrating biology and the bedside (i2b2). J Am Med Informatics Assoc. 2010;17:124–30.

[3] Athey BD, Braxenthaler M, Haas M, Guo Y. tranSMART: An Open Source and Community-Driven Informatics and Data Sharing Platform for Clinical and Translational Research. AMIA Jt Summits Transl Sci Proc AMIA Summit Transl Sci. 2013;2013:6–8.

[4] Gao J, Aksoy BA, Dogrusoz U, Dresdner G, Gross B, Sumer SO, et al. Integrative analysis of complex cancer genomics and clinical profiles using the cBioPortal. Sci Signal.

2013;6.

[5] Herzinger S, Gu W, Satagopam V, Eifes S, Rege K, Barbosa-Silva A, et al. SmartR: An open-source platform for interactive visual analytics for translational research data. Bioinformatics. 2017;33.

[6] Shiny. https://shiny.rstudio.com2018 [accessed 2018 Mar 6].

[7] Plot.ly. https://plot.ly2018 [accessed 2018 Mar 6].

[8] Bokeh. https://bokeh.pydata.org2018 [accessed 2018 Mar 6].

[9] Grinberg M. Flask Web Developement. O'Reilly. 2014.

[10] Urbanek S. Rserve – A Fast Way to Provide R Functionality to Applications. PROC 3RD Int Work Distrib Stat Comput (DSC 2003), ISSN 1609-395X, EDS KURT HORNIK, FRIEDRICH LEISCH ACHIM Zeil 2003 (HTTP//ROSUDAORG/RSERVE. 2003.

[11] Redis. https://redis.io/2018 [accessed 2018 Mar 6].

[12] Celery. http://www.celeryproject.org/2018 [accessed 2018 Mar 6].

[13] RabbitMQ. https://www.rabbitmq.com/2018 [accessed 2018 Mar 6].

[14] Hail. https://github.com/hail-is/hail2018 [accessed 2018 Apr 19].

[15] Ada. https://ada.parkinson.lu2018 [accessed 2018 Mar 6].

[16] Cortez P, Cerdeira A, Almeida F, Matos T, Reis J. Modeling wine preferences by data mining from physicochemical properties. Decis Support Syst. 2009;47:547–53.

[17] Webpack. https://webpack.github.io/2018 [accessed 2018 Mar 6].

[18] Babel. https://babeljs.io2018 [accessed 2018 Mar 6].

[19] Vue.js. https://vuejs.org/2018 [accessed 2018 Mar 6].

[20] Vuex. https://vuex.vuejs.org/en/2018 [accessed 2018 Mar 6].

[21] NPM. https://www.npmjs.com/2018 [accessed 2018 Mar 6].

[22] PyPI. https://pypi.python.org/pypi2018 [accessed 2018 Mar 6].

[23] Docker Hub. https://www.docker.com/2018 [accessed 2018 Apr 19].

[24] Reese W. Nginx: The High-performance Web Server and Reverse Proxy. Linux J. Houston, TX: Belltown Media; 2008;2008.

[25] Gunicorn. http://gunicorn.org/2018 [accessed 2018 May 1].

Click here to download Figure Figure 1 -Architecture.png

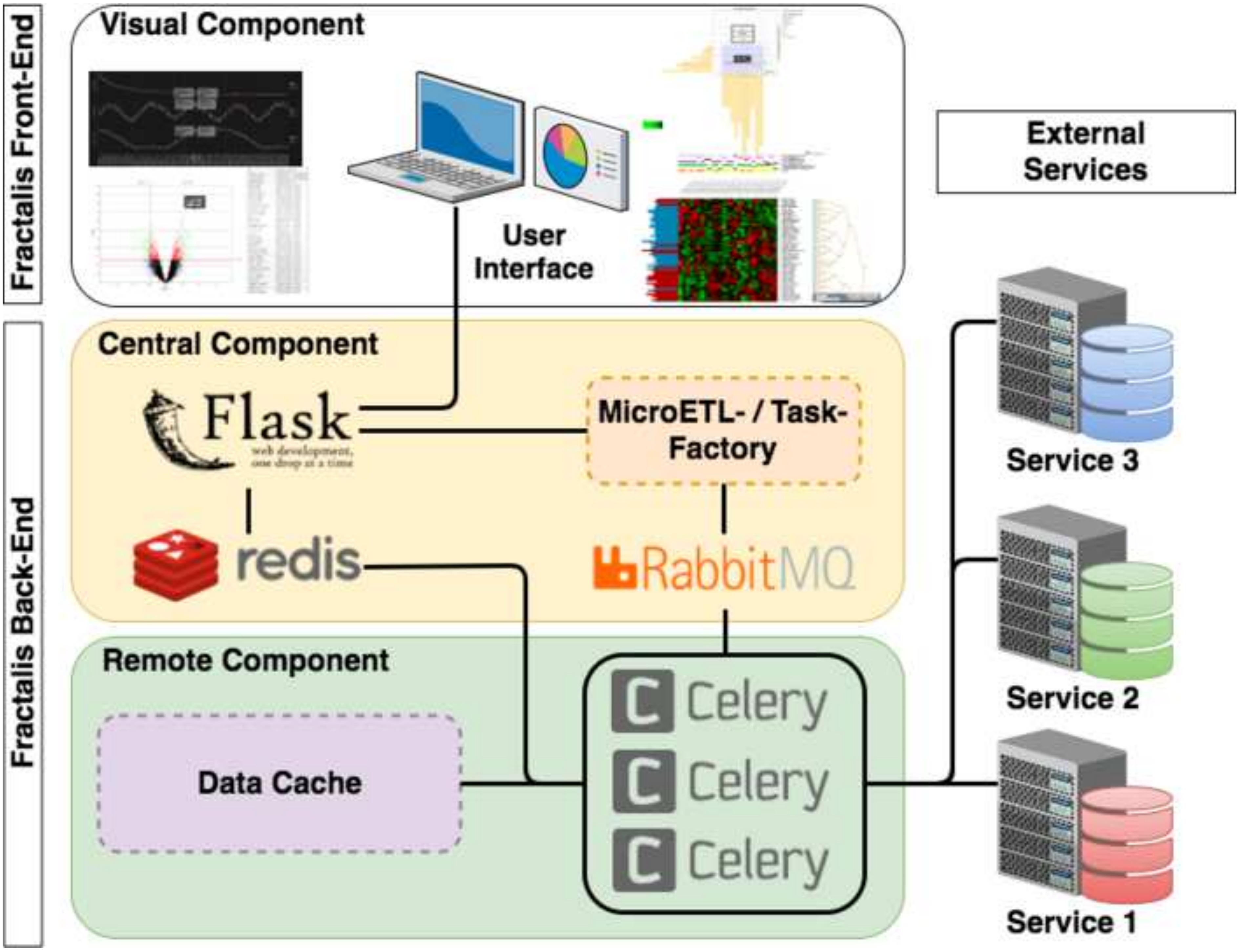

Click here to download Figure Figure 2 - Fractalis in Ada.png

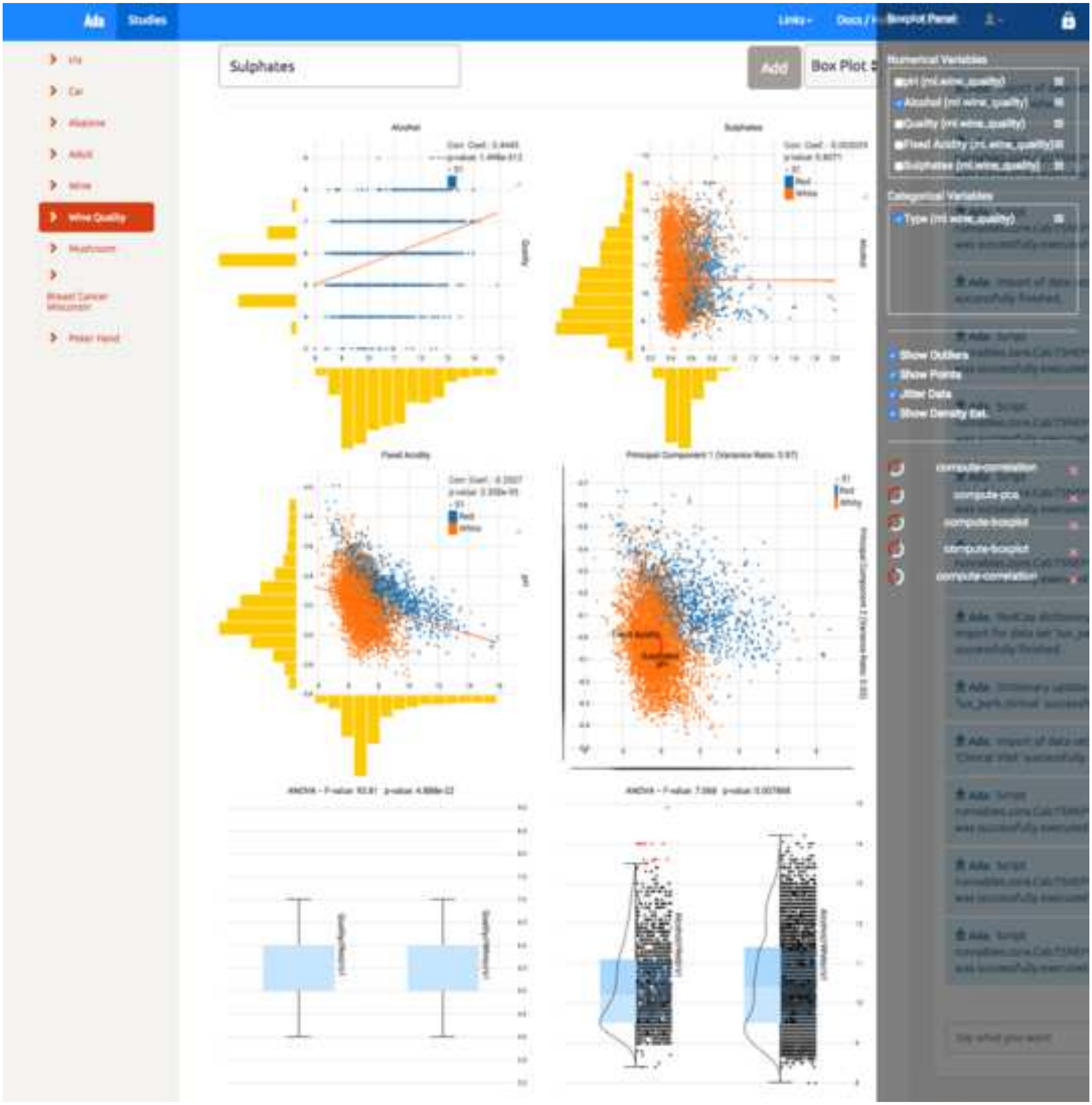

Click here to download Figure Figure 3 -Welcome Page.png

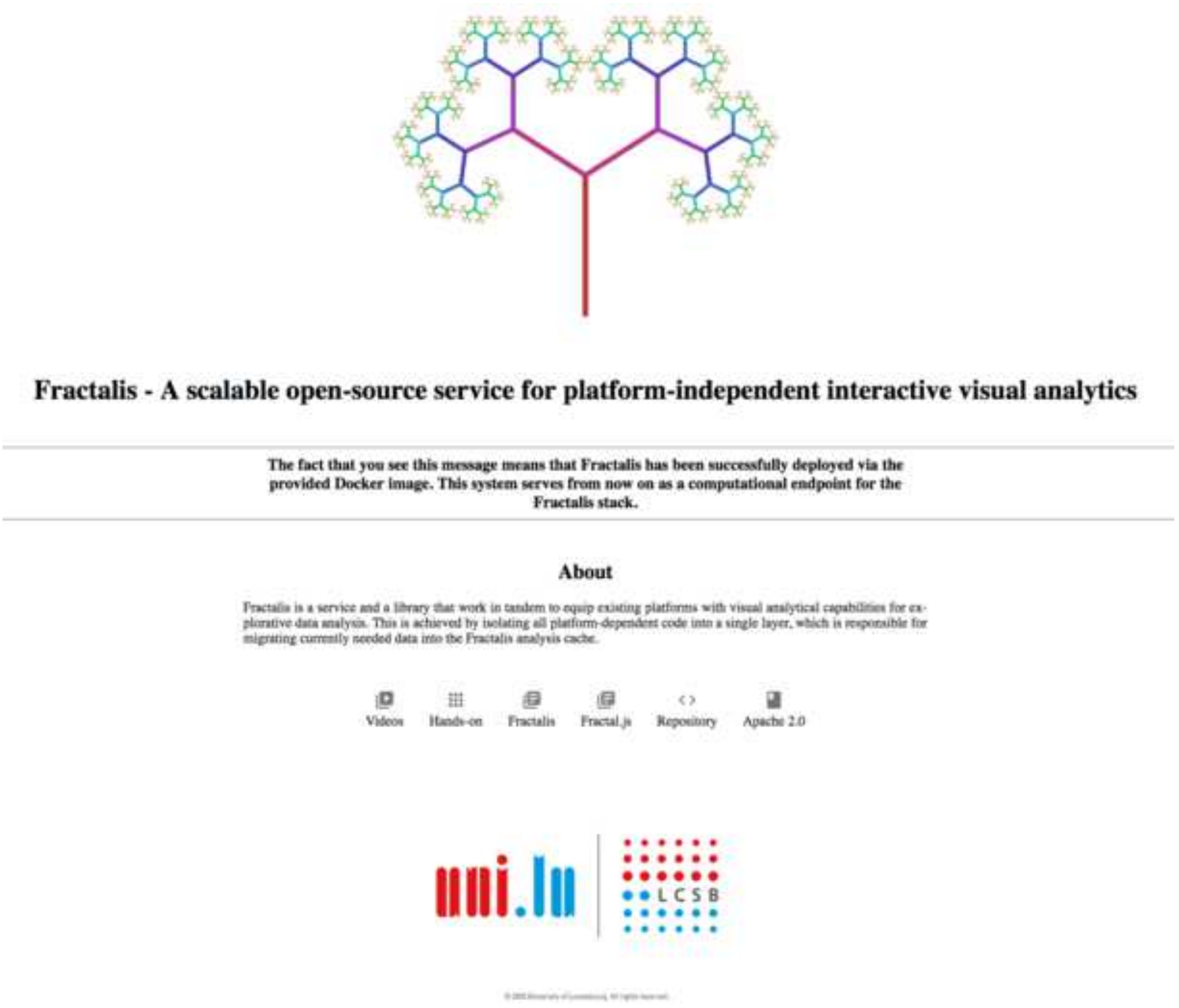

Click here to access/download
Supplementary Material
Supplementary Material.docx
