# Supplementary material for: Fractalis: a scalable open-source service for platform-independent interactive visual analysis of biomedical data
Source: Gigascience. 2018 Aug 27;7(9):giy109. doi: 10.1093/gigascience/giy109 (PMC6143733; doi:10.1093/gigascience/giy109)
Supplement: GIGA-D-18-00166_Revision_1.pdf [file giy109_giga-d-18-00166_revision_1.pdf]

GigaScience

Fractalis: A scalable open-source service for platform-independent interactive visual analysis of biomedical data

--Manuscript Draft--

Manuscript Number:	GIGA-D-18-00166R1
Full Title:	Fractalis: A scalable open-source service for platform-independent interactive visual analysis of biomedical data
Article Type:	Technical Note
Funding Information:	Fonds Nationale de la Recherche (FNR) Luxembourg (NCER13/BM/11264123) — Not applicable
Abstract:	Background: Translational research platforms share the aim to promote a deeper understanding of stored data by providing visualization and analysis tools for data exploration and hypothesis generation. However, such tools are usually platform-bound and are not easily reusable by other systems. Furthermore, they rarely address access restriction issues when direct data transfer is not permitted. In this article we present an analytical service that works in tandem with a visualization library to address these problems.<br>Findings: Using a combination of existing technologies and a platform-specific data abstraction layer we developed a service that is capable of providing existing web-based data warehouses and repositories with platform-independent visual analytical capabilities. The design of this service also allows for federated data analysis by eliminating the need to move the data directly to the researcher. Instead, all operations are based on statistics and interactive charts without direct access to the dataset.<br>Conclusion: The software presented in this article has a potential to help translational researchers achieve a better understanding of a given dataset and quickly generate new hypothesis. Furthermore, it provides a framework that can be used to share and reuse explorative analysis tools within the community.
Corresponding Author:	Sascha Herzinger<br>LCSB - University of Luxembourg<br>LUXEMBOURG
Corresponding Author Secondary Information:	
Corresponding Author's Institution:	LCSB - University of Luxembourg
Corresponding Author's Secondary Institution:	
First Author:	Sascha Herzinger, MSc
First Author Secondary Information:	
Order of Authors:	Sascha Herzinger, MSc
	Valentin Grouès, MSc
	Wei Gu, PhD
	Venkata Satagopam, MSc
	Peter Banda, PhD
	Christophe Trefois, PhD
	Reinhard Schneider, PhD
Order of Authors Secondary Information:	
Response to Reviewers:	Response to Editor and Reviewers

Powered by Editorial Manager® and ProduXion Manager® from Aries Systems Corporation

Dear Reviewers, Dear Editor,

we would like to thank you for their time and effort they put into managing and reviewing our submission. The comments are very fair and provided us with a different perspective on our work, which, in our opinion, substantially contributed to the improvement of the manuscript and the material we provide otherwise.

The major concern that you voiced was the lack of a properly documented translational research use case. We decided to use the TCGA – COAD dataset from the GDC data portal (https://portal.gdc.cancer.gov/repository) and see whether we could come to similar conclusions as this paper from 2015, which used this dataset: https://www.nature.com/articles/ncomms7879
In particular, we were interested whether we could create similar charts as seen in Figure 1, a, b, c, e (d & f are based on different datasets) with Fractalis. We added our observations to the manuscript (s. Validation by example) and recorded a new video with voice over that describes the entire process.

Below you will find our response to every point you raised during the review.

Thank you very much!

Comments Reviewer #1

“This is a well designed distributed data analysis software. The documentation (for developers) is clear on the README files of the code repository. While the authors claim translational applications, a specific example in this regard is not shown in manuscript, and this is the main reservation I have for publication. Furthermore, I did not understand exactly the type of data (translational as mentioned in the abstract) that fractalis works with. Is it NGS data, for example raw sequences, or post-bioinformatics analysis (differential RNAseq, ChipSeq etc), or it is statistical data, such as clinical trials, population studies etc. The authors need to be more specific in that regard.”

“Major revisions:”

“Demonstrate a translational case, where clearly showing the use of a dataset (there is plenty of public data available from the Short Read Archive at EMBL, or authors can use their in-house data as well). “

For the translational data analysis case, be specific and show discoveries made from the data using the visualizations that fractalis offers. Essentially demonstrate the usefulness of the systems for non-developers / end-users / researchers, who are the intended beneficiaries for the software.”

> We have addressed these points, as mentioned in the introduction above, by using a Nature Communications publication and the publicly available TCGA – COAD dataset. We have documented this process in the manuscript and in a new video and think this will make the usefulness for researchers clear.

“Discuss performance, for example, how big was the dataset, what was the computational time required (discuss capacity of the server used). Also especially given that this is a distributed system, what was the latency between the distributed servers. For example if the authors used a fractalis backend within a node running on a university in the UK and the fractalis front end was at their site in Luxemburg, would there be significant latency ? I am not expecting this to be a globally distributed system, but within the EU it should be applicable. My suggestion if the authors do not have any collaborators on a remote site to setup a backend fractalis node, is to use an instance on the Amazon Web Services which I believe have their data centers in the UK or Ireland.”

> A very good suggestion. You’re not the first person to voice your concerns regarding the latency regarding the distributed setup or overhead introduced by the stack. We added a new chapter “Benchmarks” to our manuscript that discusses this in detail. Short summary: A setup within Europe is more than feasible and in our tests even

Powered by Editorial Manager® and ProduXion Manager® from Aries Systems Corporation

transatlantic. We did however not go into detail about hardware resources and computational time required. The workers execute known statistical algorithms in python or R. Measuring this would be no different from measuring the performance of R or Python, which is not within the scope of our manuscript. There is however the potential for a speed-up if an algorithm is rewritten to profit from the horizontal scaling Fractalis provides via Celery.

“While there is clear documentation for developers installing the software on the code repository, there is a lack of documentation for developers on how to load data. Do they need to be in a specific format before loaded to Redis, and what format is this. What kind of translational data are supported, NGS read data, clinical data, others. As mentioned above a specific example of analysis with datasets from public or in-house repositories would cover this.”

> We added a new document to the repository [1] to cover this in detail. For the manuscript, the TCGA – COAD use case/paragraph/video should give the reader an initial idea about the supported data types, as you mentioned.

[1] https://git-r3lab.uni.lu/Fractalis/fractalis/blob/master/fractalis/data/README.md

“Similarly for the end users while there are demo videos available, they seem to only be generic demos of the visualizations / graphics (without any narration). Excellent examples to update the paper in regards to this, can be found at the tutorials of the Galaxy project https://vimeo.com/galaxyproject, which besides the narrative also provide specific details of data analysis / discoveries that can be performed with the platform. If not a narrative video, a detailed user manual with screen shots of the different steps can work as well.”

> Done! We recorded a new voiced video (s. suppl. mat.) that describes exactly how to reproduce the steps described in the new paragraph “Validation by example” in the manuscript. The narration should cover a large part of the functionality currently available to the user.

Minor revisions:

“The source code repository seems to be a GitHub hosted on the authors institutional server. While I trust that the authors will keep their server running , given that this is an open source project, it should be also hosted at the standard place for all open-source projects such as GitHub.com (which also guarantees that the code will be accessible in the long term)”

> The code is now mirrored at https://github.com/LCSB-BioCore

Comments Reviewer #2

“Herzinger et al. have created Fractalis, an open-source service to help promote interoperability and robust visual analytics of biomedical data. Overall, the manuscript is well written. While I am not a software developer expert (see Software comments), this does seem like a very well thought-out, robustly constructed service pipeline that takes advantage of powerful services. I do, however, have some concerns over the project's overall applicability to biomedical data science. I have outlined my feedback into major and minor points for the manuscript and software.”

“Manuscript major points”

“My major concern with the paper is that it seems too focused on the software development aspects of Fractalis, and less on its utility in biomedical data science. While the software descriptions and information are extremely useful, and absolutely should be in the manuscript, I think there should be more parts devoted to utility in biomedical data science, both in the software demo itself as well as in the manuscript. Overall, I think there should be some more both examples and descriptions of possible utilities as well as explicit use cases. The dataset that the authors include for demo visualization is great for basic software illustration but I do not think sufficient for a biomedical data science platform. There are plenty of open-source data sets in

resources such as GEO that could suffice. It would be great if the authors could demonstrate an actual use case using biomedical data.”

> We have addressed this issue, as mentioned in the introduction above, by using the TCGA – COAD dataset and a Nature Communications publication. There is now a new chapter in the manuscript (“Validation by example”) alongside a new video in the supplementary materials that should demonstrate potential use cases and the usefulness for researchers.

“There should also be more explicit instructions on how Fractalis can be used with biomedical datasets (i.e., uploading/linking data, see Software comments).”

> Regarding the uploading/downloading of data, we added a new documentation to [1] on the code repository that explains this process in detail. As mentioned above, we also recorded a voiced video that should cover large parts of the functionality Fractalis offers to the end-user.

[1] https://git-r3lab.uni.lu/Fractalis/fractalis/blob/master/fractalis/data/README.md

“By the end of the Introduction/Background, there should be a clear statement on how Fractalis can enhance data analysis capabilities (see minor points for more details).”

> We’ve added several statements on how we believe Fractalis can help other researchers/research groups at the end of the Background. Your other points regarding the Background paragraph have been addressed as well (s. below).

“Manuscript minor points”

“The authors may want to take into account the following considerations:”

“Overall:
Please be consistent with visualize vs. visualise, based on journal specifications.”

> Done!

1 . Abstract, line 35: what does "bringing analysis to the data" mean?

> We rephrased that part. It should be clearer now.

“2. Background, line 48: I would replace "has to" with "should".”

> Done!

“3. Background, line 55: hypothesis —> hypotheses”

> Done!

“4. Background, lines 70-71: while I absolutely agree that utilizing multiple various different packages is often problematic, there are tools and methods that exist to utilize the packages together that I think you should mention. For instance, there are a wealth of resources that integrate plotly and R shiny (e.g., https://plot.ly/r/shiny-tutorial/).“

> That’s indeed a very powerful combination and these tools are outstanding in what they do, but we are addressing the problem at a much higher level. Shiny for instance is difficult to integrate into existing applications by better means than iframes [1]. Fractalis on the other hand is very easy to integrate with virtually any web application. Operating at such a high level of course comes at the cost of easy customizability. We rephrased the second part of the Background to explain this better and combined this narration-wise with your other comment regarding the web applications from the Ma'ayan lab.

[1] https://stackoverflow.com/questions/15592144/how-to-integrate-r-shiny-into-current-application

“5. Background, line 73: this is an important point regarding data restriction issue of patient data. I would explicitly refer to the fact that data samples from patients often contain PHI. Another thing to keep in mind, and maybe discuss, is that there are services, such as AWS, that are beginning to support for PHI (https://aws.amazon.com/blogs/security/tag/protected-health-information/).”

> Good catch, we now explicitly mention PHI.

“6. Background (general): As mentioned, one benefit of docker technology is the fact that it can control for versioning, which plagues many studies using biomedical data sets. I would reference this study which describes the issues nicely (https://www.nature.com/articles/nbt.3780)”

> This is a very important point and we fully agree that docker can help with this issue, but this is not what we’re using it for in this manuscript, as we’re not publishing any dataset. We use Docker because (especially) R packages are famous for code changes without version number change. Additionally, some of them are very difficult to install, due to various system dependencies. With Docker we can ensure that the user/sysadmin has a very pleasant time setting this stack up (as you have done!) and we, as developers, can be sure they run the same code than us, making bugs easier to reproduce and fix.

“7. Background (general): There are many other biomedical data visualization tools and software that are not discussed. While it is obviously not possible and beyond the scope of an introduction to discuss all of them, I would consider referencing some of the tools that come from the Ma'ayan lab (http://labs.icahn.mssm.edu/maayanlab/resources/).”

> We added two references to these tools in the Background and included some additional description. This is a very useful reference because it shows the use of high-level web applications for explorative data analysis.

“8. Findings, line 105: maybe provide an example of the "internal standard formats"?”

> Done! We added some information in the manuscript. Additional more detailed information will be maintained in the developer documentation [1].

[1] https://git-r3lab.uni.lu/Fractalis/fractalis/blob/master/fractalis/data/README.md#internal-formats

“9. Findings, line 128/131: in regards to enabling analysis of extremely large data sets, please refer to the related Major point. Additionally, you might want to provide a one sentence example of this type of analysis”

> We added some examples in the text. In fact, we have rewritten this paragraph to make this and the point below clearer.

“10: Findings, line 129: phenotypical —> phenotype. Also, what is meant by this? In my experience, the phenotype data is not often large, i.e. containing covariate data which is orders of magnitude smaller than genetic data (unless of course there are millions of samples and/or many features).”

> Fractalis is aimed at small and medium sized datasets containing phenotype and omics data (e.g. mRNA expression matrix or miRNA quantification data as in our demonstrated example). There are several tools out there who try to solve large scale genotype analysis like Hail. In the use case we describe here Fractalis can do the phenotype/omics analysis and combine the results with Hails output in a unified visualization. We have rewritten that paragraph to make that clearer.

“11. The visualization library, line 144-146: I think there needs to be more explicit examples in this section overall (at least one specific example of a visualization).”

> Done! We added a small paragraph that refers to Figure 2 & 3 and list several examples.

Powered by Editorial Manager® and ProduXion Manager® from Aries Systems Corporation

“12. The visualization library, line 151: which "publicly available dataset"?”

> This refers to the [16] in the sentence. We rephrased it to make it clearer.

“13. The visualization library, line 164: I would change the arrangement of this sentence to: "This is very useful for explorative analysis, because …"”

> Done!

“14. Outlook (overall): I would provide (or reiterate if some are added in earlier sections) explicit details/examples of how Fractalis can support the field biomedical data science. For instance, how it could be added to consortia workflows, public pipelines/software, etc. “

> We added a paragraph to “Discussion and outlook” to cover this topic.

“Software”

“As I am not a seasoned software developer, I cannot expertly review on much of the back-end functionality. Overall, it seems like a very solid and well put-together pipeline. Hopefully my comments might enhance its usability for the public. I was able to successfully download and install Fractalis on my local machine. The process and instructions were very straightforward and the supplementary materials were exceptionally helpful (having instructional videos is also very nice)”

> Thank you very much for your kind words. Your feedback is highly appreciated and will help us to improve the manuscript and documentation. In particular we are glad to see that you managed to run Fractalis on your own system. Reproducibility and ease of use is very important to us.

“Major points:”

“1. I would alter the "select box" framework of the front end. Instead of having these "boxes" pre-set. I would have a function to iteratively add boxes, i.e. "Add plot" button. “

> We modified the demonstration page and added the possibility for the user to create their own layout. Please note that this is only a basic example of how the Fractalis API can be used. Other platforms might create an entirely different layout with Fractalis (e.g. Figure 2 in manuscript).

2. This may completely my misunderstanding, but I am unsure of how to upload data to Fractalis (I also didn't see a video for it). Apologies if I am confused, but it would be helpful to more clearly explain this.

> No need to apologize, Reviewer #1 asked something similar, so the documentation regarding this point was indeed lacking. We added a new README [1] that explains the implementation details of MicroETLs and added a link to it from the main README. This should make the process a lot clearer. Manual uploading data into Fractalis is possible but our demonstration page does not permit this.

[1] https://git-r3lab.uni.lu/Fractalis/fractalis/blob/master/fractalis/data/README.md

“Minor points:”

“1. I would add some of the information in the supplementary materials to the Fractalis homepage (or in a Help link).”

> We moved most of the information of the supplementary materials to [1] and added a link to this on the “main” README, which is directly accessible from the homepage.

[1] https://git-r3lab.uni.lu/Fractalis/fractalis/blob/master/docker/README.md

“2. In the readme, I would state (maybe with a screenshot?) where the docker-ip

	address can be found.”<br><br>> Unless someone uses docker-machine, the IP should always be 127.0.0.1/localhost. For docker-machine users we added the command to get the IP to the README and the supplementary materials.<br><br>“3. The Wiki link in the sidebar is not functional.”<br><br>> This link works only in our intranet. It does not contain any information relevant for public audience. As per request by the other reviewer we now mirror all content on GitHub for general public access, where this link has been removed.<br><br>“4. If you prefer to keep the "select box", I would indicate somewhere which "box" is currently selected”<br><br>> We implemented what you suggested in Software.Major.1., so this point should be solved.<br><br>“5. Consider adding an interactive "tutorial" function like with IntroJS (http://shiny.rstudio.com/articles/js-introjs.html). For instance, I didn't realize at first the awesome potential for zooming in, which also manipulates the other plots”<br>> We are glad to hear you like this functionality! We are currently considering adding guided tours to the supported services (e.g. i2b2-tranSMART, which itself is still in beta and changing frequently). As soon as the influx of new features will settle we will likely come back to IntroJS or similar solutions.<br><br>“6. I would consider moving "Hands-on" button before "Videos" and someone more clearly indicate that this begins the "actual" service.”<br><br>> We moved the button (now called “Demo”) and added changed the target page to indicate that more clearly.
Additional Information:	
Question	Response
Are you submitting this manuscript to a special series or article collection?	No
**Experimental design and statistics**<br><br>Full details of the experimental design and statistical methods used should be given in the Methods section, as detailed in our Minimum Standards Reporting Checklist. Information essential to interpreting the data presented should be made available in the figure legends.<br><br>Have you included all the information requested in your manuscript?	Yes
**Resources**<br><br>A description of all resources used, including antibodies, cell lines, animals and software tools, with enough	Yes

information to allow them to be uniquely identified, should be included in the Methods section. Authors are strongly encouraged to cite Research Resource Identifiers (RRIDs) for antibodies, model organisms and tools, where possible.<br><br>Have you included the information requested as detailed in our Minimum Standards Reporting Checklist?	
**Availability of data and materials**<br><br>All datasets and code on which the conclusions of the paper rely must be either included in your submission or deposited in publicly available repositories (where available and ethically appropriate), referencing such data using a unique identifier in the references and in the "Availability of Data and Materials" section of your manuscript.<br><br>Have you have met the above requirement as detailed in our Minimum Standards Reporting Checklist?	Yes

Manuscript with DOI
Click here to view linked References
Click here to access/download;Manuscript;Manuscript with DOI.docx

Fractalis: A scalable open-source service for platform-independent interactive visual analysis of biomedical data

Sascha Herzinger sascha.herzinger@uni.lu (correspondence)
Valentin Grouès valentin.groues@uni.lu
Wei Gu wei.gu@uni.lu
Venkata Satagopam venkata.satagopam@uni.lu
Peter Banda peter.banda@uni.lu
Christophe Trefois christophe.trefois@elixir-luxembourg.org
Reinhard Schneider reinhard.schneider@uni.lu

Luxembourg Centre for Systems Biomedicine, University of Luxembourg, Belvaux, Luxembourg.

Abstract

Background: Translational research platforms share the aim to promote a deeper understanding of stored data by providing visualization and analysis tools for data exploration and hypothesis generation. However, such tools are usually platform-bound and are not easily reusable by other systems. Furthermore, they rarely address access restriction issues when direct data transfer is not permitted. In this article we present an analytical service that works in tandem with a visualization library to address these problems.

Findings: Using a combination of existing technologies and a platform-specific data abstraction layer we developed a service that is capable of providing existing web-based data warehouses and repositories with platform-independent visual analytical capabilities. The design of this service also allows for federated data analysis by eliminating the need to move the data directly to the researcher. Instead, all operations are based on statistics and interactive charts without direct access to the dataset.

Conclusion: The software presented in this article has a potential to help translational researchers achieve a better understanding of a given dataset and quickly generate new hypothesis. Furthermore, it provides a framework that can be used to share and reuse explorative analysis tools within the community.

Keywords

visualization, visual analytics, translational research, explorative analysis, federated analysis, web service

Background

In the field of translational research, we are facing an ever-growing amount of pre-clinical, clinical, OMICS, and mobile-sensor data that should be considered as a whole to understand the bigger picture of underlying diseases and biological processes. A platform that is able to store, link, and analyze the different data formats in an integrative manner is of urgent need. In recent years several tools [1] have emerged that attempt to solve these issues by providing a framework with standardized formats and tools for *data-driven* statistical analysis. Often performed before the time-consuming and computationally expensive *hypothesis-driven* analysis, the data-driven analysis helps researchers achieve a better understanding of the data and subsequently filter and generate new hypotheses. Some known examples of such translational research platforms are i2b2 [2], tranSMART [3], and cBioPortal [4]. These and other related platforms all share similar core functionalities that are responsible for analysis and visualization. Usually these internal analytical systems are complex constructs that require high maintenance due to changing data structures and requirements. This also explains why most, if not all, translational research platforms implement their own version of such systems. Statistical analysis scripts in those implementations usually make strong assumptions about the given data format, and the visual counterparts make similar strong assumptions about the user interface (UI), of which they are part of. This makes the implementation in a given platform substantially easier, but at the same time highly platform-dependent, and in most cases unusable by other services. SmartR [5] is a concrete example for such a visual analytical system. It equips tranSMART with modern and interactive data analysis tools, but suffers from the mentioned platform-dependency, which makes the integration into other services very difficult. Shiny [6], Plotly [7], and Bokeh [8] are popular tools to address some aspects of this issue. They all make interactive visual analytics accessible to researchers with limited resources and in some cases can even be combined, as is the case with Shiny and Plotly. These tools operate at a lower level which makes them customizable, but, in the case of data analysis,

difficult to share and reuse across different existing web applications with different data formats and APIs. Furthermore, none of the mentioned software tools directly addresses the data access restriction issue that often accompanies patient studies containing protective health information (PHI). Another approach is to provide researchers with a higher level solution in form of web applications with well-defined input formats, as shown in [9] and [10], that operate on a specific set of problems. This is a very powerful approach, because it shifts the focus of the researcher to the data analysis instead of having to worry about data formats, analysis code and APIs.

Fractalis is such a web application with focus on general hypothesis generation, data exploration, scalability, and ease of integration with existing platforms. It is capable of equipping existing translational research platforms with a powerful visualization component and an analytical system with federated data analysis abilities. Modern web technologies enable a dynamic and modern experience for the user, while a powerful distributed job pipeline ensures that the service is scalable and performant. Furthermore, the isolation of platform specific code into a single layer makes it possible to extend Fractalis with support for virtually any translational research platform. This can lead to a significant reduction of resources invested in developing own visual analytical solutions.

Findings

Building an external service for distributed data analysis alone would not solve the problem of platform-dependency. APIs (Application Programming Interfaces) and data formats are likely unique to a given platform, so analysis scripts and visualizations based on the returned data would not be easily reusable. Instead we split up the solution into two components. One is a web service that is capable of extracting data from foreign APIs and transforming them into an internal standard format, so given analysis scripts would always operate the same. The other

component is a library that acts as a communication channel between the user interface (UI) of the supported platform and the Fractalis back-end. It is also largely responsible for the visualization of the analysis results.

The service

We used a Python web framework called Flask [11] as a base for our service. The main reason for that choice is the ability to use Python and R (via rpy2) natively for statistical computations. This also reduces the complexity of the application and massively improves the debugging capabilities by eliminating the need for an additional service like RServe [12]. To avoid tight coupling with foreign platforms we introduced a new concept called *Micro-ETL* within our service. Unlike usual ETLs (Extract, Transform, Load) that migrate large parts of a database, these Micro-ETLs only migrate data that are currently required. The knowledge of what is required is relayed to the Fractalis service via a Javascript library located within the foreign UI. Once this information reaches the service, a Micro-ETL factory decides which implementation can handle the migration based on the given information. What follows are the three major steps of every ETL:

- The **e**xtraction of the data is usually done via REST API but can also involve other processes or protocols. Micro-ETLs contain all knowledge necessary to communicate with a given API or extract data by other means.
- The **t**ransformation is the key to platform independence. It ensures that all incoming data are transformed to one of the internal standard formats (currently numerical data, categorical data, and array data), that makes all available analysis scripts reusable by other services that use Fractalis with similar data.

- In the loading step, the data are written to a non-persistent cache, whose location is tracked by Redis [13]. This avoids unnecessary data extraction in subsequent tasks. This is very important, given the fast-paced exploration that we want to provide.

Once the data are cached, we can perform statistical analyses on them and send the results, be it a HTML document, an image, or complex statistics to the Javascript library for further processing and visualization.

Some important challenges that visual analytics tools currently face are scalability in terms of parallel distributed job execution, federated analysis, and handling very large genomic data sets. In the following we introduce the Fractalis technology stack and describe how it handles these tasks.

The MicroETL and analysis stack mentioned before is supported by Celery [14] with RabbitMQ [15] as a message broker and Redis as result and metadata store. A schematic illustration of how these services are interconnected can be seen in Figure 1, where it is shown that the back-end part of Fractalis is separated into a central and remote component. Celery

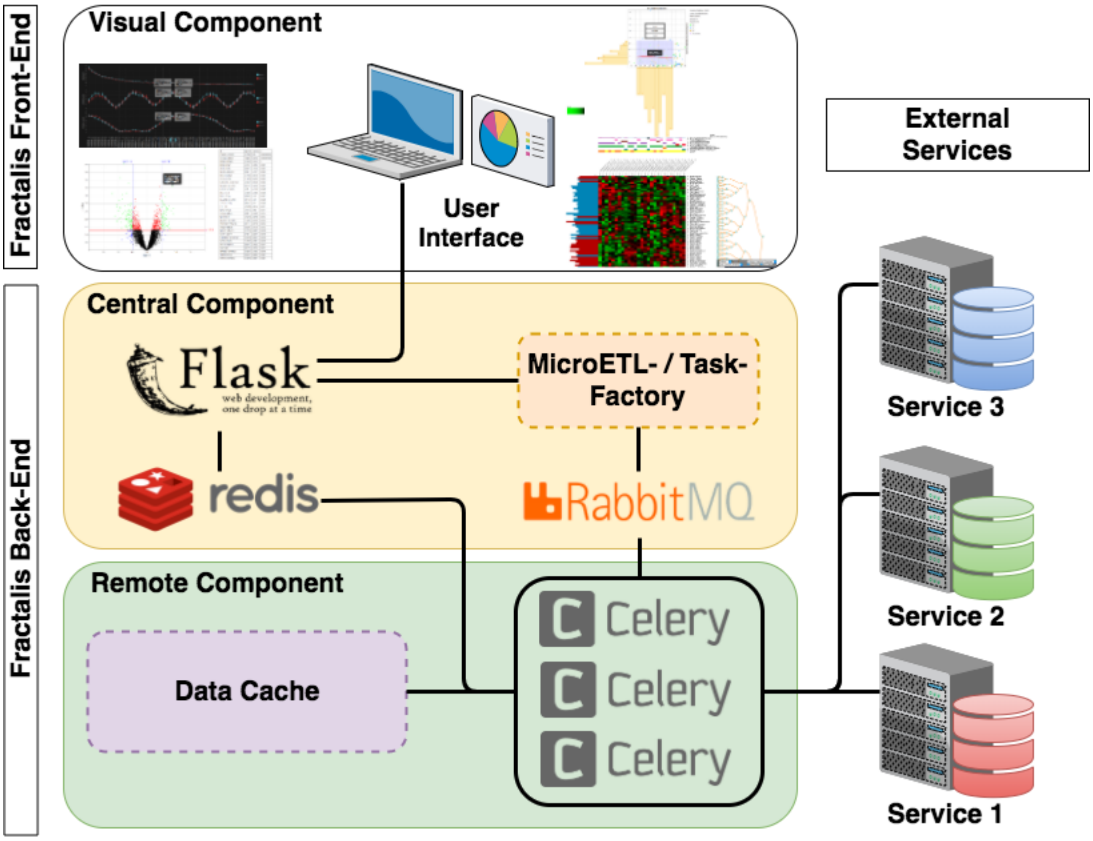

Figure 1 The Fractalis stack. Shown is a schematic view of the three major components: 1) The visual component, which resides in the web browser and interacts with the user. 2) The central server component which manages the application states and handles job distribution. 3) The remote server component, which can be deployed remotely and handles the majority of the application workload.

allows us to spawn many computational nodes (workers) on different remote machines in order to move most of the workload out of the web service itself and enable support for a very large number of parallel requests by many users. One can also observe that the data cache resides in the remote component, which has no link to the central component at all. This design supports the federated analysis paradigm because it allows us to deploy workers in restrictive environments, where they can perform or relay analysis requests and only return statistics/results, not the data itself, to the central Redis result store, and subsequently be visualized.

The very same concept also enables the combination of small/medium sized phenotype/omics statistical analyses with large scale genotype analyses, e.g. PCA, MDR, GWAS, and QTL. In such a scenario, Fractalis can handle the phenotype/omics data and relay analysis requests to frameworks like Hail [16] that can analyse large scale genotype data. Fractalis can then

combine these results in a single visualization, which is often necessary to fully understand biological processes.

Developing new Micro-ETLs or analytics within Fractalis permits, but does not enforce, usage of the more advanced functionalities of the computational stack. For instance, it is possible to write a fully independent Python script that will be executed on a remote Celery worker without ever having heard of the technology. However, a more knowledgeable developer might want to queue several subsequent jobs or parallelize certain parts of the analytical process using the more advanced Celery interface. This is achieved by a design pattern known as *factory method*, which we used several times within the application, to improve pluggability of new scripts and ETLs.

The visualization library

The purpose of the front-end component is to provide a simple API that allows the integration into existing user interfaces, and to render visualizations based on the statistics computed by the Fractalis service. In Figure 2 and 3 one can see several examples of such visualizations: a scatter plots with correlation analysis and linear regression, a PCA, box plots with one-way ANOVA, a volcano plot with differential expression analysis, and a survival analysis with Kaplen Meier or Nelson Aalen estimator.

The API is usually connected to platform specific tools for patient subset selection and other methods for selecting variables of interest. This information is sent to the Fractalis service, where they trigger MicroETLs to prepare data for the analysis cache. How such an integration can look like is shown in Figure 2. Here can be seen how we included Fractalis into another web-based data platform called Ada [17] to display statistics for a publicly available wine quality dataset (see [18]). To avoid potential conflicts with other libraries and the name scope of the parent application we used native ES6/Javascript components in combination with

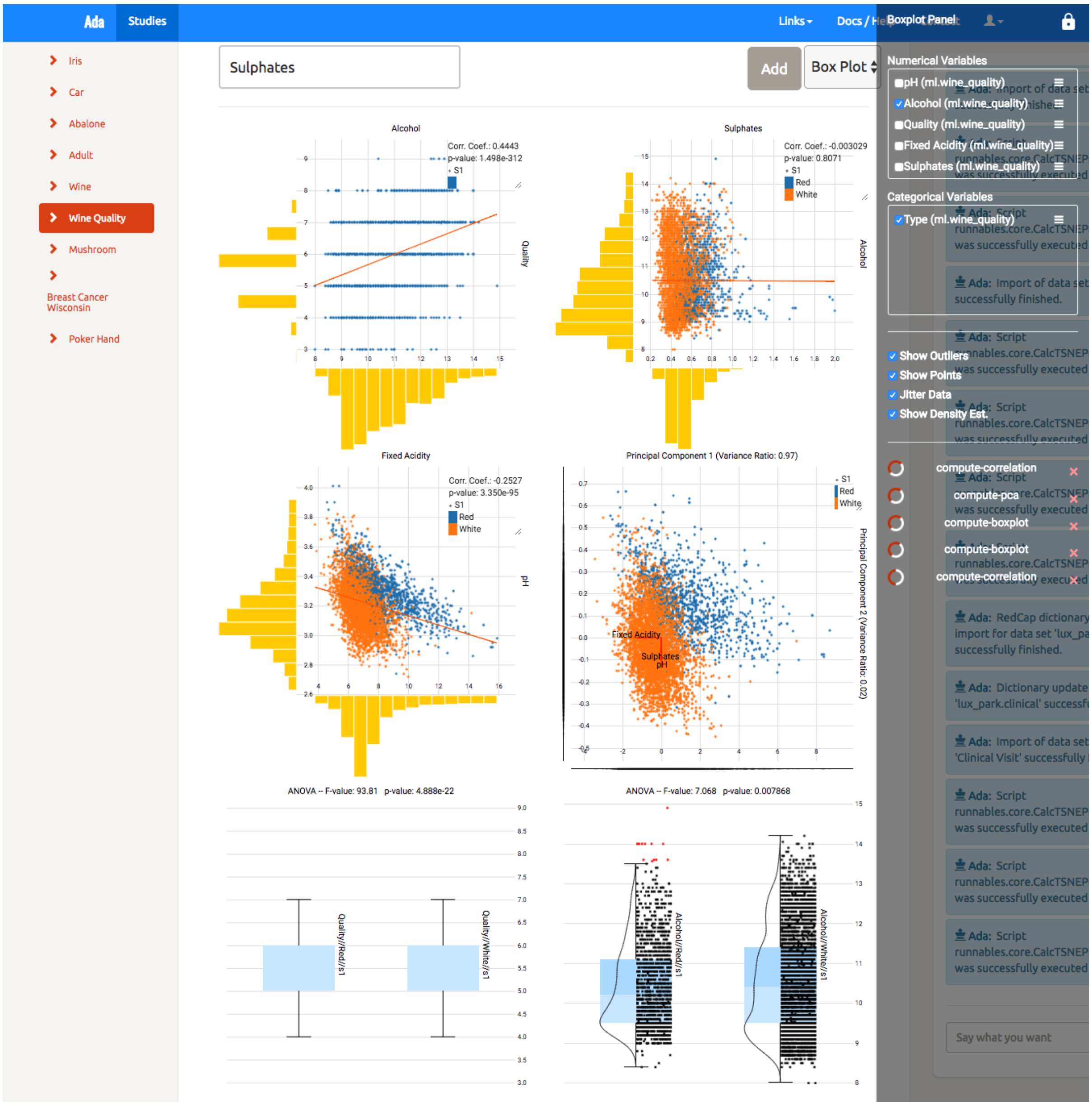

Figure 2 Fractalis in Ada. Shown is a self-hosted instance of Ada using Fractalis to display several statistics for the selected dataset. Notable is the native look of Fractalis within the existing user interface, making the integration almost completely invisible to the user.

webpack [19], which compiles the entire project into a single scoped Javascript file. Cross-browser compatibility and support for new or experimental features is ensured by Babel [20]. At the time of writing of this manuscript, modern versions of Firefox, Chrome, and Safari are all tested and supported. The charts have been created using Vue.js [21] together with a wide range of helper libraries. Reasons for this choice are the good documentation and the unopinionated nature of the framework, allowing for contributions by every moderate experienced Javascript developer. We also extended Vue.js with Vuex [22] for the ability to

have cross-component states. This is useful for mirroring the state of the server-side session or enabling the reaction of several components to a single event. In the case of Fractalis, we used this technology to connect all charts with each other in a way such that a selection within a single chart ("brushing") would inform all listeners about this event and subsequently trigger a re-computation and re-rendering of the entire view. This is very useful for explorative analysis because it allows the researcher to select groups of interest and see instantly how statistics change in comparison. Simple examples for this are the comparison between case and control group, or the exclusion of subjects with an age lower than 30. To fully understand how this technology works we recommend a look into the videos and guides that are linked in the supplementary materials.

Ensuring continuous reproducibility

Several measures have been taken to ensure reproducibility and ease of deployment, when working with the Fractalis service. First of all, the code is properly documented and tested with roughly 250 unit and functional tests at the time of writing. These tests are executed for every code submission to our self-hosted GitLab repository and for every release. The release process is completely automated and requires no manual interaction. By pushing a new tag to the code repository, the continuous integration (CI) pipeline is instructed to build a new test environment, run all tests within this environment, and publish the build artifacts to their respective public repositories if all tests pass. Altogether the artifacts are published to three repositories, namely NPM [23] for the Javascript library, PyPI [24] for the Python package, and Docker Hub for the Docker images [25]. To even further simplify the deployment of Fractalis we made use of the Docker Compose technology, which manages the service setup and network including Redis, RabbitMQ, Nginx [26], Gunicorn [27], the Fractalis web service,

and the Fractalis worker. In fact, the setup has become so simple that we encourage the readers to follow the instructions in the supplementary materials and deploy Fractalis themselves.

Validation by example

We extensively discussed the technological aspects of the service presented in this manuscript but so far have not mentioned a specific translational research use case. To demonstrate the usefulness of the tools described in this manuscript, we selected a publication (Bu et al. 2015) [28] with several plots that are based on analyses of the TCGA – COAD [29] dataset. In particular, we will focus on the miRNA quantification data and clinical data. All TCGA datasets are public and can be downloaded without registration from their repositories. The mentioned plots can be found in [28] Figure 1 a, b, c, e:

1. a volcano plot based on a differential expression analysis of the microRNA quantification data
2. a MA plot based on the same data
3. a box plot with a group test for the difference between early and late stage expression of a certain microRNA
4. a survival analysis based on the same microRNA between high and low read count of the same microRNA.

In the same order than above, the purpose of these analyses are:

1. the discovery of up or downregulated microRNA with high significance
2. making sure that microRNAs of potential interest are present in sufficiently abundance
3. testing whether there is a significant difference between early and late stage cancer for a certain microRNA
4. testing whether the number of reads for a certain microRNA is correlated with the survival time of the patient

To validate our analyses pipelines we created every chart with Fractalis and checked if we came to similar conclusions than the authors of the publication. It should be noted at this point that the TCGA – COAD dataset substantially grew (now 465 samples) since the paper was published. Additionally, the authors did not describe their methods in detail, making a perfect

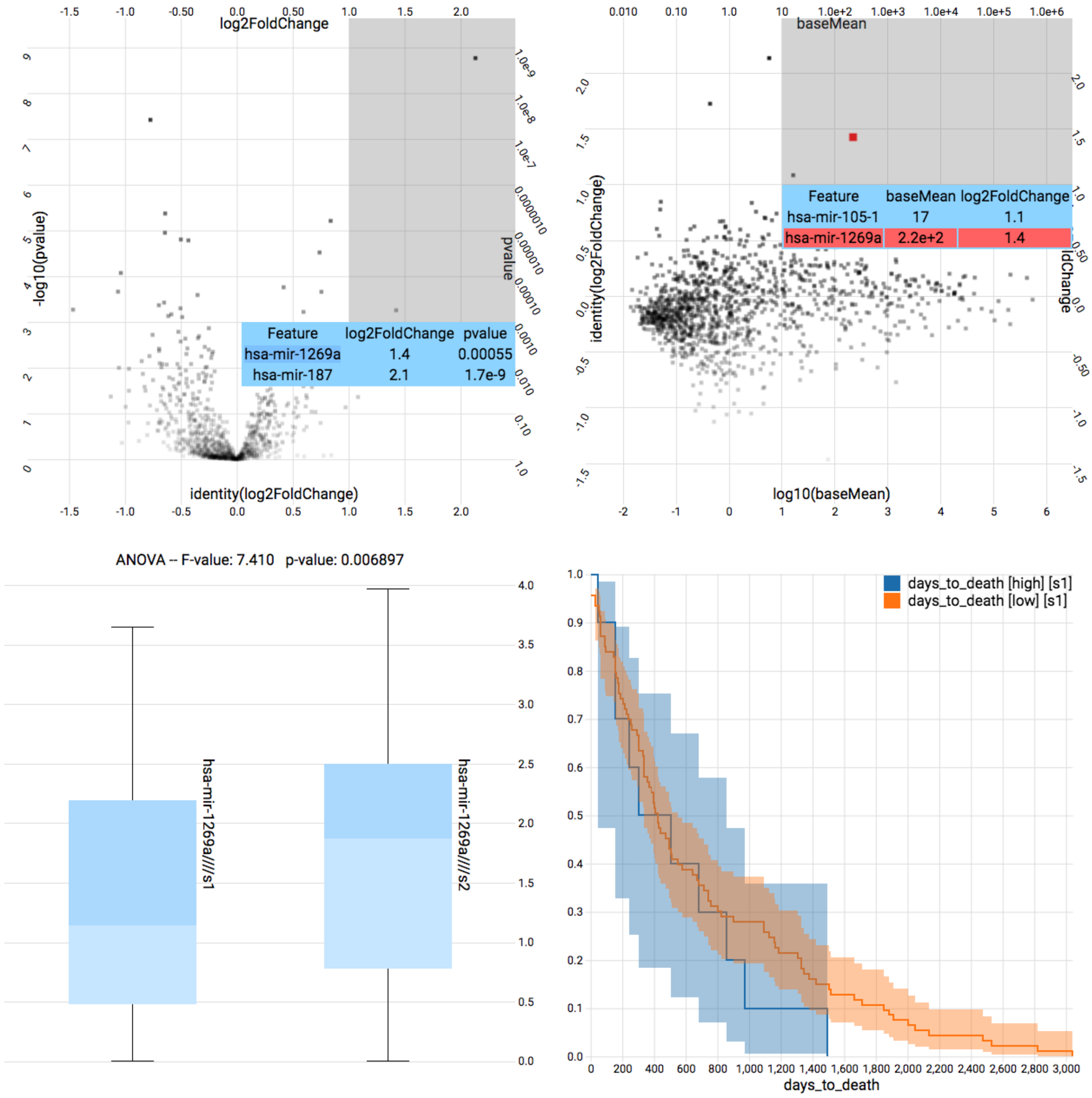

Figure 3 Fractalis pipeline demonstration. Shown are four Fractalis charts that show statistics based on the TCGA – COAD dataset. From left to right, top to bottom: a) A volcano plot using results of the R package DESeq2 b) A MA plot using results of the R package DESeq2 c) Box plots and a one-way ANOVA group test d) A survival plot using the Kaplan Meier estimator. a)-c) compare early stage cancer with late stage cancer. d) compares high with low read count of has-mir-1269a.

reproduction very difficult, if not impossible. Nevertheless, the analyses results should be similar enough to make a valid comparison. Figure 3 shows the result of this comparison. We recorded a video of the process of creating these charts (s. supplementary materials) and

included the dataset in the docker image, so interested readers may try to create the charts on their own. In the following we describe our observations in detail.

First, we created the volcano plot (s. Figure 3 a)) by plotting the log2 fold change against the negative log10 fold-change. These statistics where obtained by using the R DESeq2 package. We selected the sector within the chart with p <= 0.01 and log2 (fold change) >= 1.0, which revealed 3 microRNAs, namely has-mir-1269a (p = 0.00055, log2(FC) = 1.4), has-mir-187 (p = 1.7e-7, log2(FC) = 2.1), and has-mir-934 (p = 0.0035, log2(FC) = 1.7). Subsequently, we created a MA plot (s. Figure 3 b)) using the same results, but this time plotting log10(baseMean) against log2(FC) and selected the sector within the chart with baseMean >= 10 and log2(FC) >= 1.0. This revealed has-mir-105-1 (baseMean = 17, log2(FC) = 1.1) and has-mir-1269a (baseMean = 2.2e+2, log2(FC) = 1.4). Intersecting this result with the previous one leaves has-mir-1269a as the only microRNA with sufficient abundancy, significance, and noteworthy fold change between early and late stage cancer. So far, our results are in alignment with the findings reported by the authors. We singled out the has-mir-1269a row from the data matrix and compared the two groups within a box plot chart (s. Figure 3 c)) with log10 transformed data. The one-way ANOVA reported F = 7.410 with p = 0.0069. Our boxplots are almost identical to the reported ones but our group test has a slightly better p-value, likely due to the additional samples in the dataset. Finally, we compared samples with high has-mir-1269a read count (#reads > 1500) with low has-mir-1269a read count (#reads < 1500) by performing a survival analysis with a Kaplan Meier estimator. We only included patients with an observed death event in both groups, but our results in rough agreement with the observations of Bu et al., who likely considered the entire cohort. It should be noted here that the group with high read has-mir-1269a count is very small, which is highlighted by the very large confidence intervals. All shown charts can be generated in a matter of seconds or minutes (differential expression analysis is computationally expensive) within Fractalis.

Latency Benchmarks

The Fractalis back-end can be horizontally scaled by using a distributed worker architecture. As mentioned further above, this particular point also allows for federated analysis, which is why it is of particular interest how well this architecture performs when there is a large physical distance between the central and the remote nodes. For this purpose, we used Google Cloud to deploy worker nodes in the UK and the US, with a central node in Germany and the actual user interface in Luxembourg. To get a baseline, we deployed the same setup in our intranet, so we can approximate the introduced latency. To avoid a potential bias due to different hardware specifications, we limited this test to the execution of a simple correlation analysis with linear regression between two variables within the TCGA – COAD dataset from above. In this way, only the network latency between the different services would be measured, not how well the CPU and RAM perform. It should also be noted that the current front-end is polling in intervals for the result, so the measured latency will be higher than the actual latency. In Table 1 we listed the outcome of the 3 different setups. Unsurprisingly, the intranet deployment is the fastest with an average of 88ms past between submitting an analysis and receiving the results. This is the most common form of deployment and will be sufficient for any potential use case targeted by Fractalis. Moving the central component to a server in Frankfurt – Germany and the worker to a server in London – UK resulted in an expected increase of the latency. With 207ms on average this latency is hardly noticeable, making a large distributed network within Europe more than feasible. Moving the worker to a server in Carolina – USA introduced a noticeable lag of on average 792ms. While this is a 9-fold increase in comparison to our intranet baseline, the delay is still in the sub-second space. Most explorative analyses with a moderately sized dataset will run for several seconds, making a sub-second delay in most cases neglectable.

Worker Location	Ping	1st Meas.	2nd Meas.	3rd Meas.	Avg.
Intranet	< 1ms	92ms	84ms	88ms	88ms
London - UK (Google Cloud)	20ms	222ms	200ms	199ms	207ms
Carolina - US (Google Cloud)	102ms	794ms	794ms	789ms	792ms

Table 1 Fractalis distributed pipeline benchmark. The table shows the time past between submitting an analysis and receiving the results. All results include the time needed to prepare the data for analysis, the computation of the correlation statistics, the sending of the results, and the latency/overhead introduced by the communication between the service components. The Ping column shows the base latency by pinging the server from our location in Luxembourg.

Discussion and outlook

In this manuscript we presented a framework for explorative visual analysis of biomedical data. Major features include easy integration into almost all existing translational research platforms and the heavily distributed architecture that permits high scalability and enables analysis federation functionality. For platforms with little or no explorative data analysis, Fractalis is a real alternative to developing an own solution and can save development resources and give researchers access to many useful tools for hypothesis generation. Furthermore, own visualizations and analyses can easily be shared with other researchers, even if the underlying data warehouse platform is different.

The authors demonstrated the hypothesis generation capabilities by quickly generating several charts and comparing them with an existing publication and recorded their work for educational purpose.

A few standard analyses have been included in Fractalis to showcase the software and gather an initial user base. Some examples are survival analyses, box plots, scatter plots with correlation analysis and linear regression, volcano plots and heat maps with differential

expression analysis performed by the R packages limma and DESeq2, and principle component analyses.

New developments will take user feedback into account and prioritize the implementation of much needed analyses and features. In a similar fashion, support for new platforms in form of new MicroETLs will follow. At the time of writing, Fractalis is being integrated into three different platforms:

1. tranSMART 17.1 including the new data model, API, and UI
2. Ada [manuscript in preparation], an internally developed data integration service
3. i2b2-tranSMART [manuscript in preparation], a platform developed by the recently merged i2b2 Foundation and tranSMART Foundation. Note: This platform is *very* different from 1.

Further future developments might include a stand-alone version of Fractalis, which runs on the computer of the researcher to permit uploading and analyzing local files via the user interface instead of extracting it from an external service.

Availability and requirements

Project name: Fractalis

RRID: SCR_016362

Project home page: https://fractalis.lcsb.uni.lu/

Operating systems: All Docker supported operating systems (e.g. most Linux distributions, MacOS, MS Windows)

Programming languages: Python, Javascript

Requirements: Python 3.6 or higher, a recent version of Chrome, Firefox, or Safari

License: Apache 2.0

Availability of supporting data

The data set supporting the results of this article is available at https://portal.gdc.cancer.gov/repository. Snapshots of the code and other supporting data is also openly available in the GigaScience repository, GigaDB [30].

List of abbreviations

API – Application Programming Interface

CI – Continuous Integration

COAD - Colon Adenocarcinoma

ETL – Extract Transform Load

PHI – Protective Health Information

TCGA – The Cancer Genome Atlas

UI – User Interface

Ethics approval and consent to participate

Not applicable

Consent for publication

Not applicable

Competing interests

The authors declare that they have no competing interests.

Funding

Acknowledgement is made for support by the Fonds Nationale de la Recherche (FNR) Luxembourg, through the National Centre of Excellence in Research (NCER) on Parkinson's disease, NCER13/BM/11264123.

This work was partially funded through the contribution of the Luxembourg Ministry of Higher Education and Research towards the Luxembourg ELIXIR Node.

Authors' contributions

SH planned and executed the project. VG contributed their knowledge to the architecture. PB is a very early adopter of Fractalis and their feedback influenced the development process. CT provided the necessary infrastructure for a large-scale setup and helped with the deployment. WG, VS, RS are senior researchers that helped to understand the current translational research landscape and its challenges. All authors read the manuscript and provided feedback.

Acknowledgements

The authors would like to thank their colleagues and the Reproducible Research Results (R3) team of the Luxembourg Centre for Systems Biomedicine for support of the project and for promoting reproducible research.

References

[1] Canuel V, Rance B, Avillach P, Degoulet P, Burgun A. Translational research platforms integrating clinical and omics data: A review of publicly available solutions. Brief Bioinform. 2015;16:280–90.

[2] Murphy SN, Weber G, Mendis M, Gainer V, Chueh HC, Churchill S, et al. Serving the enterprise and beyond with informatics for integrating biology and the bedside (i2b2). J Am Med Informatics Assoc. 2010;17:124–30.

[3] Athey BD, Braxenthaler M, Haas M, Guo Y. tranSMART: An Open Source and Community-Driven Informatics and Data Sharing Platform for Clinical and Translational Research. http://www.ncbi.nlm.nih.gov/pubmed/24303286%5Cnhttp://www.pubmedcentral.nih.gov/articlerender.fcgi?artid=PMC3814495AMIA Jt Summits Transl Sci Proc AMIA Summit Transl Sci [Internet]. 2013;2013:6–8.

[4] Gao J, Aksoy BA, Dogrusoz U, Dresdner G, Gross B, Sumer SO, et al. Integrative analysis of complex cancer genomics and clinical profiles using the cBioPortal. Sci Signal. 2013;6.

[5] Herzinger S, Gu W, Satagopam V, Eifes S, Rege K, Barbosa-Silva A, et al. SmartR: An open-source platform for interactive visual analytics for translational research data. Bioinformatics. 2017;33.

[6] Shiny. https://shiny.rstudio.com2018 [accessed 2018 Mar 6].

[7] Plot.ly. https://plot.ly2018 [accessed 2018 Mar 6].

[8] Bokeh. https://bokeh.pydata.org2018 [accessed 2018 Mar 6].

[9] Wang Z, Monteiro CD, Jagodnik KM, Fernandez NF, Gundersen GW, Rouillard AD, et al. Extraction and analysis of signatures from the Gene Expression Omnibus by the crowd. Nat Commun. 2016;

[10] Wang Z, Lachmann A, Keenan AB, Ma'ayan A. L1000FWD: fireworks visualization of drug-induced transcriptomic signatures. http://dx.doi.org/10.1093/bioinformatics/bty060Bioinformatics [Internet]. 2018;34:2150–2.

[11] Grinberg M. Flask Web Developement. O'Reilly. 2014;

[12] Urbanek S. Rserve – A Fast Way to Provide R Functionality to Applications. PROC 3RD Int Work Distrib Stat Comput (DSC 2003), ISSN 1609-395X, EDS KURT HORNIK, FRIEDRICH LEISCH ACHIM Zeil 2003 (HTTP//ROSUDAORG/RSERVE. 2003.

[13] Redis. https://redis.io/2018 [accessed 2018 Mar 6].

[14] Celery. http://www.celeryproject.org/2018 [accessed 2018 Mar 6].

[15] RabbitMQ. https://www.rabbitmq.com/2018 [accessed 2018 Mar 6].

[16] Hail. https://github.com/hail-is/hail2018 [accessed 2018 Apr 19].

[17] Ada. https://ada.parkinson.lu2018 [accessed 2018 Mar 6].

[18] Cortez P, Cerdeira A, Almeida F, Matos T, Reis J. Modeling wine preferences by data mining from physicochemical properties. Decis Support Syst. 2009;47:547–53.

[19] Webpack. https://webpack.github.io/2018 [accessed 2018 Mar 6].

[20] Babel. https://babeljs.io2018 [accessed 2018 Mar 6].

[21] Vue.js. https://vuejs.org/2018 [accessed 2018 Mar 6].

[22] Vuex. https://vuex.vuejs.org/en/2018 [accessed 2018 Mar 6].

[23] NPM. https://www.npmjs.com/2018 [accessed 2018 Mar 6].

[24] PyPI. https://pypi.python.org/pypi2018 [accessed 2018 Mar 6].

[25] Docker Hub. https://www.docker.com/2018 [accessed 2018 Apr 19].

[26] Reese W. Nginx: The High-performance Web Server and Reverse Proxy. http://dl.acm.org/citation.cfm?id=1412202.1412204Linux J [Internet]. Houston, TX: Belltown Media; 2008;2008.

[27] Gunicorn. http://gunicorn.org/2018 [accessed 2018 May 1].

[28] Bu P, Wang L, Chen KY, Rakhilin N, Sun J, Closa A, et al. miR-1269 promotes metastasis and forms a positive feedback loop with TGF-β. Nat Commun. 2015;

[29] Tomczak K, Czerwińska P, Wiznerowicz M. The Cancer Genome Atlas (TCGA): an immeasurable source of knowledge. Contemp Oncol. Termedia Publishing; 2015;19:A68.

[30] Herzinger S, Grouès V, Gu W, Satagopam V, Banda P, Trefois C, Schneider R: Supporting data for "Fractalis: A scalable open-source service for platform-independent interactive visual analysis of biomedical data" GigaScience Database. 2018. http://dx.doi.org/10.5524/100497.

Figure 1

Click here to access/download;Figure;Figure 1 - Architecture.png

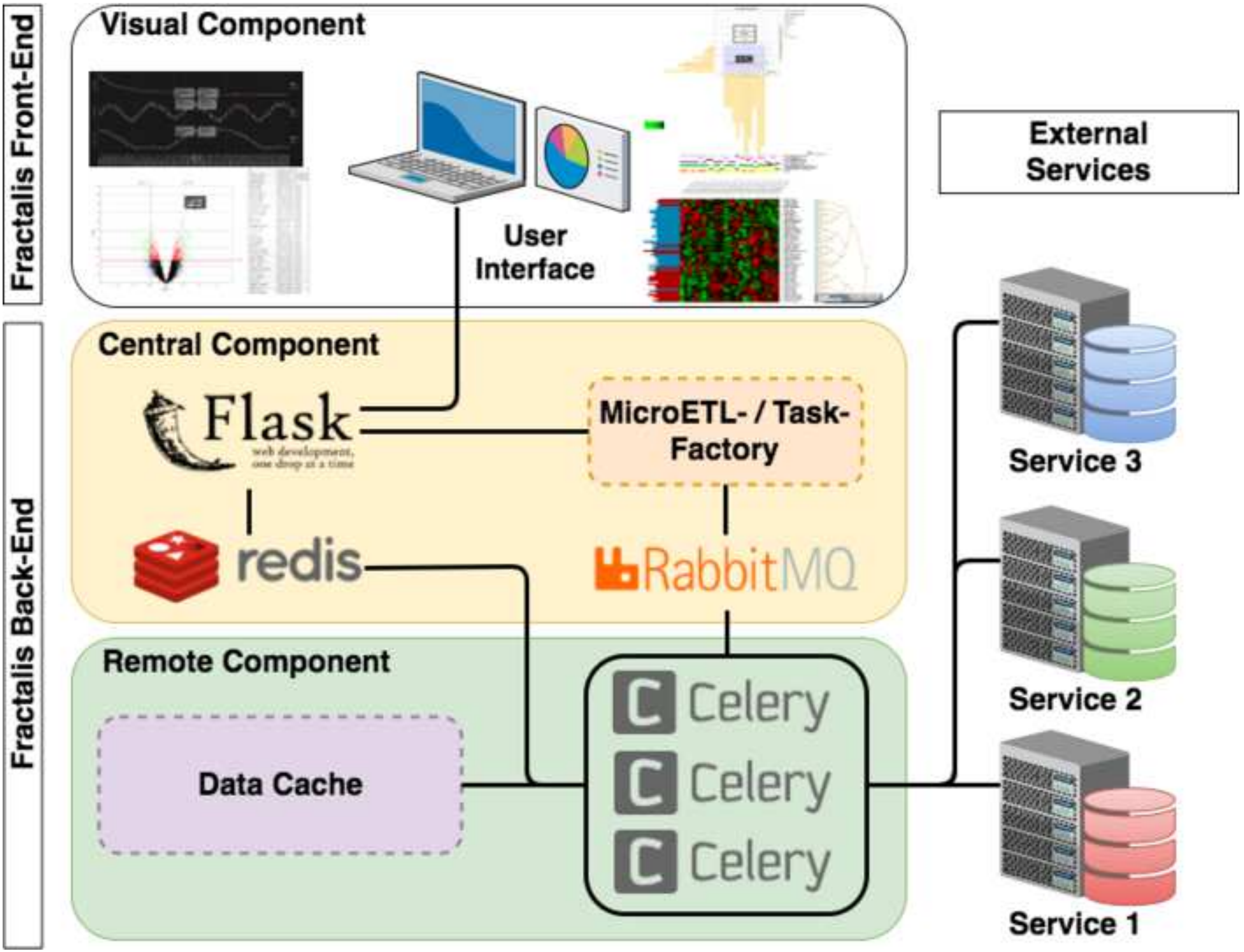

Figure 2

Click here to access/download;Figure;Figure 2 - Fractalis in Ada.png

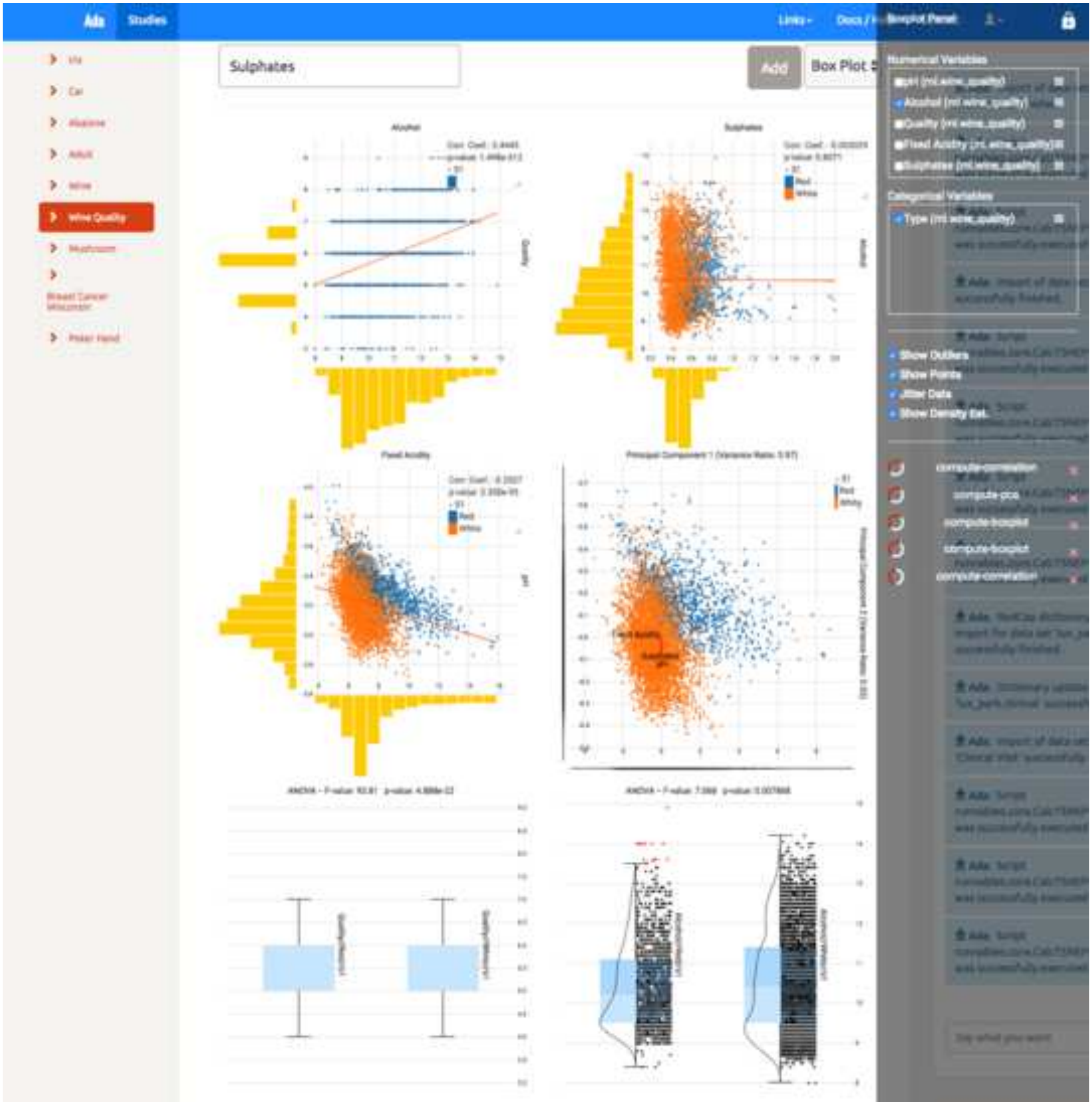

Figure 3

Click here to access/download;Figure;Figure 3 - Validation.png

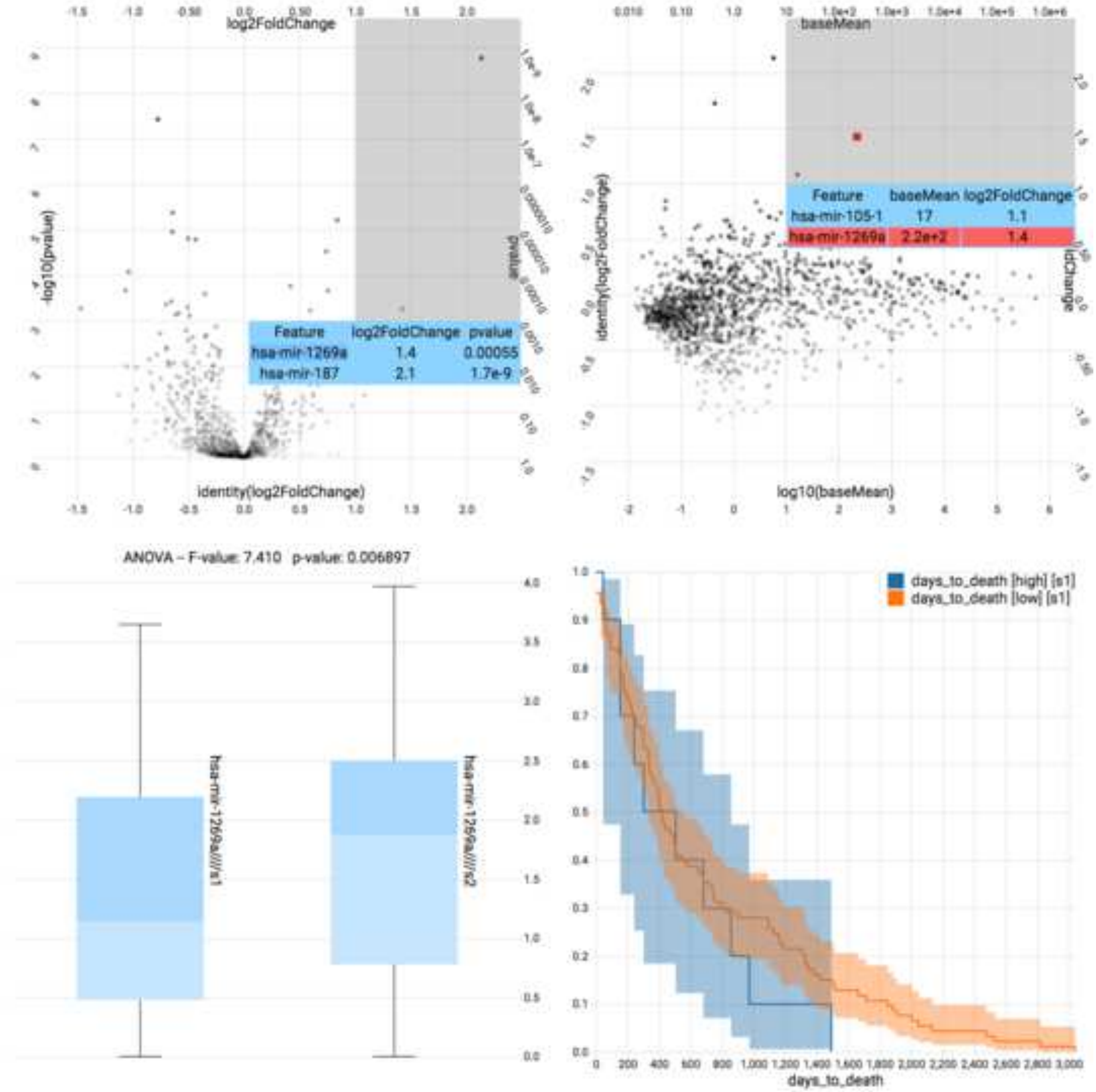

Click here to access/download
Supplementary Material
Supplementary Material.docx
