# Supplementary material for: Fractalis: a scalable open-source service for platform-independent interactive visual analysis of biomedical data
Source: Gigascience. 2018 Aug 27;7(9):giy109. doi: 10.1093/gigascience/giy109 (PMC6143733; doi:10.1093/gigascience/giy109)
Supplement: Supplement File [file giy109_supplement_file.docx]

**Supplementary Material**

**Fractalis: A scalable open-source service for platform-independent interactive visual analysis of biomedical data**

Sascha Herzinger, Valentin Grouès, Wei Gu, Venkata Satagopam, Peter Banda, Christophe Trefois, Reinhard Schneider

**Content**

1. Video material
2. Deploying Fractalis with Docker
3. Online example

**1. Video material**

In Figure 1 in the main manuscript we showed how the integration of Fractalis in Ada looks like, but a static image does not reflect the dynamic nature of the visualisations. To highlight these parts, we recorded a video and added it to a Playlist that can be accessed by following the URL below. This playlist also contains several videos that demonstrate several other functionalities that Fractalis has to offer, including narrative examples using publicly available dataset.

<https://www.youtube.com/playlist?list=PLNvp9GB9uBmH1NNAf-qTyj_jN2aCPISFU>

Whenever additional video material will become available it will also be added to this playlist.

**2. Deploying Fractalis with Docker**

We assume that Docker and Docker-Compose are already installed on the system and are up-to-date. It is possible to check this by opening a terminal and running the following commands:

If these commands fail or if the versions are much older than the one displayed above please consult <https://docs.docker.com/install/> and <https://docs.docker.com/compose/install/> .

If docker is properly installed on your system please run the following commands:

The last command might require root access to connect with the Docker engine. Depending on your network connection, this step will take a few minutes. Once all the services are up and running you can open Chrome, Firefox, or Safari and navigate to <http://localhost> or, if you use docker-machine, to http:// + the value returned by `docker-machine ip`. If you see the Fractalis logo, your system just became a Fractalis node that can be used for statistical computation, as long as Docker is running. We tested this on several fresh installations of Linux and MacOS and experienced no issues. If this fails for you, make sure docker is properly installed and port 80 and 443 are not used by other services on your system.

In addition, we included a small stand-alone demo in every image. If you press the “Demo” icon, you will be able to try Fractalis for yourself. Please keep in mind, that this is a very basic page for demonstration and educational purpose only.

**3. Online example**

For those who do not wish to or cannot install Fractalis themselves we provide a self-hosted deployment at the URL below. Please keep in mind that despite our best efforts and monitoring this service can be subject to temporary maintenance or network issues.

<https://fractalis.lcsb.uni.lu/>
