# Supplementary material for: Fractalis: a scalable open-source service for platform-independent interactive visual analysis of biomedical data
Source: Gigascience. 2018 Aug 27;7(9):giy109. doi: 10.1093/gigascience/giy109 (PMC6143733; doi:10.1093/gigascience/giy109)
Supplement: Response_to_Reviewer_Comments_Original_Submission.pdf [file giy109_response_to_reviewer_comments_original_submission.pdf]

Author's Response To Reviewer Comments

Close

Response to Editor and Reviewers

Dear Reviewers, Dear Editor,

we would like to thank you for their time and effort they put into managing and reviewing our submission. The comments are very fair and provided us with a different perspective on our work, which, in our opinion, substantially contributed to the improvement of the manuscript and the material we provide otherwise.

The major concern that you voiced was the lack of a properly documented translational research use case. We decided to use the TCGA – COAD dataset from the GDC data portal (https://portal.gdc.cancer.gov/repository) and see whether we could come to similar conclusions as this paper from 2015, which used this dataset: https://www.nature.com/articles/ncomms7879
In particular, we were interested whether we could create similar charts as seen in Figure 1, a, b, c, e (d & f are based on different datasets) with Fractalis. We added our observations to the manuscript (s. Validation by example) and recorded a new video with voice over that describes the entire process.

Below you will find our response to every point you raised during the review.

Thank you very much!

Comments Reviewer #1

“This is a well designed distributed data analysis software. The documentation (for developers) is clear on the README files of the code repository. While the authors claim translational applications, a specific example in this regard is not shown in manuscript, and this is the main reservation I have for publication. Furthermore, I did not understand exactly the type of data (translational as mentioned in the abstract) that fractalis works with. Is it NGS data, for example raw sequences, or post-bioinformatics analysis (differential RNAseq, ChipSeq etc), or it is statistical data, such as clinical trials, population studies etc. The authors need to be more specific in that regard.”

“Major revisions:”

“Demonstrate a translational case, where clearly showing the use of a dataset (there is plenty of public data available from the Short Read Archive at EMBL, or authors can use their in-house data as well). “

For the translational data analysis case, be specific and show discoveries made from the data using the visualizations that fractalis offers. Essentially demonstrate the usefulness of the systems for non-developers / end-users / researchers, who are the intended beneficiaries for

the software.”

> We have addressed these points, as mentioned in the introduction above, by using a Nature Communications publication and the publicly available TCGA – COAD dataset. We have documented this process in the manuscript and in a new video and think this will make the usefulness for researchers clear.

“Discuss performance, for example, how big was the dataset, what was the computational time required (discuss capacity of the server used). Also especially given that this is a distributed system, what was the latency between the distributed servers. For example if the authors used a fractalis backend within a node running on a university in the UK and the fractalis front end was at their site in Luxemburg, would there be significant latency ? I am not expecting this to be a globally distributed system, but within the EU it should be applicable. My suggestion if the authors do not have any collaborators on a remote site to setup a backend fractalis node, is to use an instance on the Amazon Web Services which I believe have their data centers in the UK or Ireland.”

> A very good suggestion. You’re not the first person to voice your concerns regarding the latency regarding the distributed setup or overhead introduced by the stack. We added a new chapter “Benchmarks” to our manuscript that discusses this in detail. Short summary: A setup within Europe is more than feasible and in our tests even transatlantic. We did however not go into detail about hardware resources and computational time required. The workers execute known statistical algorithms in python or R. Measuring this would be no different from measuring the performance of R or Python, which is not within the scope of our manuscript. There is however the potential for a speed-up if an algorithm is rewritten to profit from the horizontal scaling Fractalis provides via Celery.

“While there is clear documentation for developers installing the software on the code repository, there is a lack of documentation for developers on how to load data. Do they need to be in a specific format before loaded to Redis, and what format is this. What kind of translational data are supported, NGS read data, clinical data, others. As mentioned above a specific example of analysis with datasets from public or in-house repositories would cover this.”

> We added a new document to the repository [1] to cover this in detail. For the manuscript, the TCGA – COAD use case/paragraph/video should give the reader an initial idea about the supported data types, as you mentioned.

[1] https://git-r3lab.uni.lu/Fractalis/fractalis/blob/master/fractalis/data/README.md

“Similarly for the end users while there are demo videos available, they seem to only be generic demos of the visualizations / graphics (without any narration). Excellent examples to update the paper in regards to this, can be found at the tutorials of the Galaxy project https://vimeo.com/galaxyproject, which besides the narrative also provide specific details of data analysis / discoveries that can be performed with the platform. If not a narrative video, a detailed user manual with screen shots of the different steps can work as well.”

> Done! We recorded a new voiced video (s. suppl. mat.) that describes exactly how to reproduce the steps described in the new paragraph “Validation by example” in the manuscript. The narration should cover a large part of the functionality currently available to

the user.

Minor revisions:

“The source code repository seems to be a GitHub hosted on the authors institutional server. While I trust that the authors will keep their server running , given that this is an open source project, it should be also hosted at the standard place for all open-source projects such as GitHub.com (which also guarantees that the code will be accessible in the long term)”

> The code is now mirrored at https://github.com/LCSB-BioCore

Comments Reviewer #2

“Herzinger et al. have created Fractalis, an open-source service to help promote interoperability and robust visual analytics of biomedical data. Overall, the manuscript is well written. While I am not a software developer expert (see Software comments), this does seem like a very well thought-out, robustly constructed service pipeline that takes advantage of powerful services. I do, however, have some concerns over the project's overall applicability to biomedical data science. I have outlined my feedback into major and minor points for the manuscript and software.”

“Manuscript major points”

“My major concern with the paper is that it seems too focused on the software development aspects of Fractalis, and less on its utility in biomedical data science. While the software descriptions and information are extremely useful, and absolutely should be in the manuscript, I think there should be more parts devoted to utility in biomedical data science, both in the software demo itself as well as in the manuscript. Overall, I think there should be some more both examples and descriptions of possible utilities as well as explicit use cases. The dataset that the authors include for demo visualization is great for basic software illustration but I do not think sufficient for a biomedical data science platform. There are plenty of open-source data sets in resources such as GEO that could suffice. It would be great if the authors could demonstrate an actual use case using biomedical data.”

> We have addressed this issue, as mentioned in the introduction above, by using the TCGA – COAD dataset and a Nature Communications publication. There is now a new chapter in the manuscript (“Validation by example”) alongside a new video in the supplementary materials that should demonstrate potential use cases and the usefulness for researchers.

“There should also be more explicit instructions on how Fractalis can be used with biomedical datasets (i.e., uploading/linking data, see Software comments).”

> Regarding the uploading/downloading of data, we added a new documentation to [1] on the code repository that explains this process in detail. As mentioned above, we also recorded a voiced video that should cover large parts of the functionality Fractalis offers to the end-user.

[1] https://git-r3lab.uni.lu/Fractalis/fractalis/blob/master/fractalis/data/README.md

“By the end of the Introduction/Background, there should be a clear statement on how

Fractalis can enhance data analysis capabilities (see minor points for more details).”

> We’ve added several statements on how we believe Fractalis can help other researchers/research groups at the end of the Background. Your other points regarding the Background paragraph have been addressed as well (s. below).

“Manuscript minor points”

“The authors may want to take into account the following considerations:”

“Overall:
Please be consistent with visualize vs. visualise, based on journal specifications.”

> Done!

1 . Abstract, line 35: what does "bringing analysis to the data" mean?

> We rephrased that part. It should be clearer now.

“2. Background, line 48: I would replace "has to" with "should".”

> Done!

“3. Background, line 55: hypothesis —> hypotheses”

> Done!

“4. Background, lines 70-71: while I absolutely agree that utilizing multiple various different packages is often problematic, there are tools and methods that exist to utilize the packages together that I think you should mention. For instance, there are a wealth of resources that integrate plotly and R shiny (e.g., https://plot.ly/r/shiny-tutorial/).“

> That’s indeed a very powerful combination and these tools are outstanding in what they do, but we are addressing the problem at a much higher level. Shiny for instance is difficult to integrate into existing applications by better means than iframes [1]. Fractalis on the other hand is very easy to integrate with virtually any web application. Operating at such a high level of course comes at the cost of easy customizability. We rephrased the second part of the Background to explain this better and combined this narration-wise with your other comment regarding the web applications from the Ma'ayan lab.

[1] https://stackoverflow.com/questions/15592144/how-to-integrate-r-shiny-into-current-application

“5. Background, line 73: this is an important point regarding data restriction issue of patient data. I would explicitly refer to the fact that data samples from patients often contain PHI. Another thing to keep in mind, and maybe discuss, is that there are services, such as AWS, that are beginning to support for PHI (https://aws.amazon.com/blogs/security/tag/protected-health-information/).”

> Good catch, we now explicitly mention PHI.

“6. Background (general): As mentioned, one benefit of docker technology is the fact that it can control for versioning, which plagues many studies using biomedical data sets. I would reference this study which describes the issues nicely (https://www.nature.com/articles/nbt.3780)”

> This is a very important point and we fully agree that docker can help with this issue, but this is not what we’re using it for in this manuscript, as we’re not publishing any dataset. We use Docker because (especially) R packages are famous for code changes without version number change. Additionally, some of them are very difficult to install, due to various system dependencies. With Docker we can ensure that the user/sysadmin has a very pleasant time setting this stack up (as you have done!) and we, as developers, can be sure they run the same code than us, making bugs easier to reproduce and fix.

“7. Background (general): There are many other biomedical data visualization tools and software that are not discussed. While it is obviously not possible and beyond the scope of an introduction to discuss all of them, I would consider referencing some of the tools that come from the Ma'ayan lab (http://labs.icahn.mssm.edu/maayanlab/resources/).”

> We added two references to these tools in the Background and included some additional description. This is a very useful reference because it shows the use of high-level web applications for explorative data analysis.

“8. Findings, line 105: maybe provide an example of the "internal standard formats"?”

> Done! We added some information in the manuscript. Additional more detailed information will be maintained in the developer documentation [1].

[1] https://git-r3lab.uni.lu/Fractalis/fractalis/blob/master/fractalis/data/README.md#internal-formats

“9. Findings, line 128/131: in regards to enabling analysis of extremely large data sets, please refer to the related Major point. Additionally, you might want to provide a one sentence example of this type of analysis”

> We added some examples in the text. In fact, we have rewritten this paragraph to make this and the point below clearer.

“10: Findings, line 129: phenotypical —> phenotype. Also, what is meant by this? In my experience, the phenotype data is not often large, i.e. containing covariate data which is orders of magnitude smaller than genetic data (unless of course there are millions of samples and/or many features).”

> Fractalis is aimed at small and medium sized datasets containing phenotype and omics data (e.g. mRNA expression matrix or miRNA quantification data as in our demonstrated example). There are several tools out there who try to solve large scale genotype analysis like Hail. In the use case we describe here Fractalis can do the phenotype/omics analysis and combine the results with Hails output in a unified visualization. We have rewritten that paragraph to make that clearer.

“11. The visualization library, line 144-146: I think there needs to be more explicit examples in this section overall (at least one specific example of a visualization).”

> Done! We added a small paragraph that refers to Figure 2 & 3 and list several examples.

“12. The visualization library, line 151: which "publicly available dataset"?”

> This refers to the [16] in the sentence. We rephrased it to make it clearer.

“13. The visualization library, line 164: I would change the arrangement of this sentence to: "This is very useful for explorative analysis, because …"”

> Done!

“14. Outlook (overall): I would provide (or reiterate if some are added in earlier sections) explicit details/examples of how Fractalis can support the field biomedical data science. For instance, how it could be added to consortia workflows, public pipelines/software, etc. “

> We added a paragraph to “Discussion and outlook” to cover this topic.

“Software”

“As I am not a seasoned software developer, I cannot expertly review on much of the back-end functionality. Overall, it seems like a very solid and well put-together pipeline. Hopefully my comments might enhance its usability for the public. I was able to successfully download and install Fractalis on my local machine. The process and instructions were very straightforward and the supplementary materials were exceptionally helpful (having instructional videos is also very nice)”

> Thank you very much for your kind words. Your feedback is highly appreciated and will help us to improve the manuscript and documentation. In particular we are glad to see that you managed to run Fractalis on your own system. Reproducibility and ease of use is very important to us.

“Major points:”

“1. I would alter the "select box" framework of the front end. Instead of having these "boxes" pre-set. I would have a function to iteratively add boxes, i.e. "Add plot" button. “

> We modified the demonstration page and added the possibility for the user to create their own layout. Please note that this is only a basic example of how the Fractalis API can be used. Other platforms might create an entirely different layout with Fractalis (e.g. Figure 2 in manuscript).

2. This may completely my misunderstanding, but I am unsure of how to upload data to Fractalis (I also didn't see a video for it). Apologies if I am confused, but it would be helpful to more clearly explain this.

> No need to apologize, Reviewer #1 asked something similar, so the documentation regarding this point was indeed lacking. We added a new README [1] that explains the

implementation details of MicroETLs and added a link to it from the main README. This should make the process a lot clearer. Manual uploading data into Fractalis is possible but our demonstration page does not permit this.

[1] https://git-r3lab.uni.lu/Fractalis/fractalis/blob/master/fractalis/data/README.md

“Minor points:”

“1. I would add some of the information in the supplementary materials to the Fractalis homepage (or in a Help link).”

> We moved most of the information of the supplementary materials to [1] and added a link to this on the “main” README, which is directly accessible from the homepage.

[1] https://git-r3lab.uni.lu/Fractalis/fractalis/blob/master/docker/README.md

“2. In the readme, I would state (maybe with a screenshot?) where the docker-ip address can be found.”

> Unless someone uses docker-machine, the IP should always be 127.0.0.1/localhost. For docker-machine users we added the command to get the IP to the README and the supplementary materials.

“3. The Wiki link in the sidebar is not functional.”

> This link works only in our intranet. It does not contain any information relevant for public audience. As per request by the other reviewer we now mirror all content on GitHub for general public access, where this link has been removed.

“4. If you prefer to keep the "select box", I would indicate somewhere which "box" is currently selected”

> We implemented what you suggested in Software.Major.1., so this point should be solved.

“5. Consider adding an interactive "tutorial" function like with IntroJS (http://shiny.rstudio.com/articles/js-introjs.html). For instance, I didn't realize at first the awesome potential for zooming in, which also manipulates the other plots”
> We are glad to hear you like this functionality! We are currently considering adding guided tours to the supported services (e.g. i2b2-tranSMART, which itself is still in beta and changing frequently). As soon as the influx of new features will settle we will likely come back to IntroJS or similar solutions.

“6. I would consider moving "Hands-on" button before "Videos" and someone more clearly indicate that this begins the "actual" service.”

> We moved the button (now called “Demo”) and added changed the target page to indicate that more clearly.

Close
