# Supplementary material for: Fractalis: a scalable open-source service for platform-independent interactive visual analysis of biomedical data
Source: Gigascience. 2018 Aug 27;7(9):giy109. doi: 10.1093/gigascience/giy109 (PMC6143733; doi:10.1093/gigascience/giy109)
Supplement: Reviewer_1_Report_(Original_Submission) -- Konstantinos Krampis — 05/12/2018 Reviewed [file giy109_reviewer_1_report_(original_submission).pdf]

Reviewer Report

Title: **Fractalis: A scalable open-source service for platform-independent interactive visual analysis of biomedical data**

Version: **Original Submission** **Date:** 5/12/2018

Reviewer name: **Konstantinos Krampis**

Reviewer Comments to Author:

This is a well designed distributed data analysis software. The documentation (for developers) is clear on the README files of the code repository. While the authors claim translational applications, a specific example in this regard is not shown in manuscript, and this is the main reservation I have for publication. Furthermore, I did not understand exactly the type of data (translational as mentioned in the abstract) that fractalis works with. Is it NGS data, for example raw sequences, or post-bioinformatics analysis (differential RNAseq, ChipSeq etc), or it is statistical data, such as clinical trials, population studies etc. The authors need to be more specific in that regard.Major revisions:- Demonstrate a translational case, where clearly showing the use of a dataset (there is plenty of public data available from the Short Read Archive at EMBL, or authors can use their in-house data as well).- For the translational data analysis case, be specific and show discoveries made from the data using the visualizations that fractalis offers. Essentially demonstrate the usefulness of the systems for non-developers / end-users / researchers, who are the intended beneficiaries for the software. - Discuss performance, for example, how big was the dataset, what was the computational time required (discuss capacity of the server used). Also especiallygiven that this is a distributed system, what was the latency between the distributed servers. For example if the authors used a fractalis backend within a noderunning on a university in the UK and the fractalis front end was at their site in Luxemburg, would there be significant latency ? I am not expecting this to be a globally distributed system, but within the EU it should be applicable. My suggestion if the authors do not have any collaborators on a remote site to setup a backend fractalis node, is to use an instance on the Amazon Web Services which I believe have their data centers in the UK or Ireland.- While there is clear documentation for developers installing the software on the code repository, there is a lack of documentation for developers on how to load data. Do they need to be in a specific format before loaded to Redis, and what format is this. What kind of translational data are supported, NGS read data, clinical data, others. As mentioned above a specific example of analysis with datasets from public or in-house repositories would cover this.- Similarly for the end users while there are demo videos available, they seem to only be generic demos of the visualizations / graphics (without any narration). Excellent examples to update the paper in regards to this, can be found at the tutorials of the Galaxy project https://vimeo.com/galaxyproject, which besides the narrative also provide specific details of data analysis / discoveries that can be performed with the platform. If not a narrative video, a detailed user manual with screen shots of the different steps can work as well.Minor revisions:- The source code repository seems to be a GitHub hosted on the authors institutional server. While I trust that the authors will keep their server running , given that this is an open source project, it should be also hosted at the standard place for all open-source projects such as GitHub.com (which also guarantees that the code will be accessible in the long term).

Level of Interest

Please indicate how interesting you found the manuscript: Choose an item.

Quality of Written English

Please indicate the quality of language in the manuscript: Choose an item.

Declaration of Competing Interests

Please complete a declaration of competing interests, considering the following questions:

- Have you in the past five years received reimbursements, fees, funding, or salary from an organisation that may in any way gain or lose financially from the publication of this manuscript, either now or in the future?
- Do you hold any stocks or shares in an organisation that may in any way gain or lose financially from the publication of this manuscript, either now or in the future?
- Do you hold or are you currently applying for any patents relating to the content of the manuscript?
- Have you received reimbursements, fees, funding, or salary from an organization that holds or has applied for patents relating to the content of the manuscript?
- Do you have any other financial competing interests?
- Do you have any non-financial competing interests in relation to this paper?

If you can answer no to all of the above, write 'I declare that I have no competing interests' below. If your reply is yes to any, please give details below.

No competing interests declared.

I agree to the open peer review policy of the journal. I understand that my name will be included on my report to the authors and, if the manuscript is accepted for publication, my named report including any attachments I upload will be posted on the website along with the authors' responses. I agree for my report to be made available under an Open Access Creative Commons CC-BY license (http://creativecommons.org/licenses/by/4.0/). I understand that any comments which I do not wish to be included in my named report can be included as confidential comments to the editors, which will not be published.

I agree to the open peer review policy of the journal

To further support our reviewers, we have joined with Publons, where you can gain additional credit to further highlight your hard work (see: https://publons.com/journal/530/gigascience). On publication of this paper, your review will be automatically added to Publons, you can then choose whether or not to claim your Publons credit. I understand this statement. Yes
