# Supplementary material for: Fractalis: a scalable open-source service for platform-independent interactive visual analysis of biomedical data
Source: Gigascience. 2018 Aug 27;7(9):giy109. doi: 10.1093/gigascience/giy109 (PMC6143733; doi:10.1093/gigascience/giy109)
Supplement: Reviewer_2_Report_(Original_Submission) -- Benjamin Glicksberg — 05/27/2018 Reviewed [file giy109_reviewer_2_report_(original_submission).pdf]

Reviewer Report

Title: **Fractalis: A scalable open-source service for platform-independent interactive visual analysis of biomedical data**

Version: **Original Submission** **Date:** 5/27/2018

Reviewer name: **Benjamin Glicksberg**

Reviewer Comments to Author:

Herzinger et al. have created Fractalis, an open-source service to help promote interoperability and robust visual analytics of biomedical data. Overall, the manuscript is well written. While I am not a software developer expert (see Software comments), this does seem like a very well thought-out, robustly constructed service pipeline that takes advantage of powerful services. I do, however, have some concerns over the project's overall applicability to biomedical data science. I have outlined my feedback into major and minor points for the manuscript and software.Manuscript major pointsMy major concern with the paper is that it seems too focused on the software development aspects of Fractalis, and less on its utility in biomedical data science. While the software descriptions and information are extremely useful, and absolutely should be in the manuscript, I think there should be more parts devoted to utility in biomedical data science, both in the software demo itself as well as in the manuscript. Overall, I think there should be some more both examples and descriptions of possible utilities as well as explicit use cases. The dataset that the authors include for demo visualization is great for basic software illustration but I do not think sufficient for a biomedical data science platform. There are plenty of open-source data sets in resources such as GEO that could suffice. It would be great if the authors could demonstrate an actual use case using biomedical data.There should also be more explicit instructions on how Fractalis can be used with biomedical datasets (i.e., uploading/linking data, see Software comments). By the end of the Introduction/Background, there should be a clear statement on how Fractalis can enhance data analysis capabilities (see minor points for more details).Manuscript minor pointsThe authors may want to take into account the following considerations:Overall:Please be consistent with visualize vs. visualise, based on journal specifications. 1. Abstract, line 35: what does "bringing analysis to the data" mean?2. Background, line 48: I would replace "has to" with "should".3. Background, line 55: hypothesis —> hypotheses4. Background, lines 70-71: while I absolutely agree that utilizing multiple various different packages is often problematic, there are tools and methods that exist to utilize the packages together that I think you should mention. For instance, there are a wealth of resources that integrate plotly and R shiny (e.g., https://plot.ly/r/shiny-tutorial/). 5. Background, line 73: this is an important point regarding data restriction issue of patient data. I would explicitly refer to the fact that data samples from patients often contain PHI. Another thing to keep in mind, and maybe discuss, is that there are services, such as AWS, that are beginning to support for PHI (https://aws.amazon.com/blogs/security/tag/protected-health-information/).6. Background (general): As mentioned, one benefit of docker technology is the fact that it can control for versioning, which plagues many studies using biomedical data sets. I would reference this study which describes the issues nicely (https://www.nature.com/articles/nbt.3780)7. Background (general): There are many other biomedical data visualization tools and software that are not discussed. While it is obviously not possible and beyond the scope of an introduction to discuss all of them, I would consider referencing some of the tools that come from the Ma'ayan lab (http://labs.icahn.mssm.edu/maayanlab/resources/).8. Findings, line 105: maybe provide an example of the "internal standard formats"?9. Findings, line 128/131: in regards to enabling analysis of extremely large data sets, please refer to the related Major point. Additionally, you might want to provide a one sentence example of this type of analysis10: Findings, line 129: phenotypical —> phenotype. Also, what is meant by this? In my experience, the phenotype data is not often large, i.e. containing covariate data which is orders of magnitude smaller than genetic data (unless of course there are millions of samples and/or many features).11. The visualization library, line 144-146: I think there needs to be more explicit examples in this section overall (at least one specific example of a visualization). 12. The visualization library, line 151: which "publicly available dataset"?13. The visualization library, line 164: I

would change the arrangement of this sentence to: "This is very useful for explorative analysis, because …"14. Outlook (overall): I would provide (or reiterate if some are added in earlier sections) explicit details/examples of how Fractalis can support the field biomedical data science. For instance, how it could be added to consortia workflows, public pipelines/software, etc. SoftwareAs I am not a seasoned software developer, I cannot expertly review on much of the back-end functionality. Overall, it seems like a very solid and well put-together pipeline. Hopefully my comments might enhance its usability for the public. I was able to successfully download and install Fractalis on my local machine. The process and instructions were very straightforward and the supplementary materials were exceptionally helpful (having instructional videos is also very nice).Major points:1. I would alter the "select box" framework of the front end. Instead of having these "boxes" pre-set. I would have a function to iteratively add boxes, i.e. "Add plot" button. 2. This may completely my misunderstanding, but I am unsure of how to upload data to Fractalis (I also didn't see a video for it). Apologies if I am confused, but it would be helpful to more clearly explain this.Minor points:1. I would add some of the information in the supplementary materials to the Fractalis homepage (or in a Help link).2. In the readme, I would state (maybe with a screenshot?) where the docker-ip address can be found.3. The Wiki link in the sidebar is not functional.4. If you prefer to keep the "select box", I would indicate somewhere which "box" is currently selected5. Consider adding an interactive "tutorial" function like with IntroJS (http://shiny.rstudio.com/articles/js-introjs.html). For instance, I didn't realize at first the awesome potential for zooming in, which also manipulates the other plots6. I would consider moving "Hands-on" button before "Videos" and someone more clearly indicate that this begins the "actual" service.

Level of Interest

Please indicate how interesting you found the manuscript: Choose an item.

Quality of Written English

Please indicate the quality of language in the manuscript: Choose an item.

Declaration of Competing Interests

Please complete a declaration of competing interests, considering the following questions:

- Have you in the past five years received reimbursements, fees, funding, or salary from an organisation that may in any way gain or lose financially from the publication of this manuscript, either now or in the future?
- Do you hold any stocks or shares in an organisation that may in any way gain or lose financially from the publication of this manuscript, either now or in the future?
- Do you hold or are you currently applying for any patents relating to the content of the manuscript?
- Have you received reimbursements, fees, funding, or salary from an organization that holds or has applied for patents relating to the content of the manuscript?
- Do you have any other financial competing interests?
- Do you have any non-financial competing interests in relation to this paper?

If you can answer no to all of the above, write 'I declare that I have no competing interests' below. If your reply is yes to any, please give details below.

I declare that I have no competing interests

I agree to the open peer review policy of the journal. I understand that my name will be included on my report to the authors and, if the manuscript is accepted for publication, my named report including any attachments I upload will be posted on the website along with the authors' responses. I agree for my report to be made available under an Open Access Creative Commons CC-BY license (http://creativecommons.org/licenses/by/4.0/). I understand that any comments which I do not wish to be included in my named report can be included as confidential comments to the editors, which will not be published.

I agree to the open peer review policy of the journal
